# Causal Approach to Determining the Environmental Risks of Seabed Mining

**DOI:** 10.1021/acs.est.1c01241

**Published:** 2021-06-21

**Authors:** Laura Kaikkonen, Inari Helle, Kirsi Kostamo, Sakari Kuikka, Anna Törnroos, Henrik Nygård, Riikka Venesjärvi, Laura Uusitalo

**Affiliations:** † Ecosystems and Environment Research Programme, Faculty of Biological and Environmental Sciences, University of Helsinki, 00014 Helsinki, Finland; ‡ Helsinki Institute of Sustainability Science (HELSUS), University of Helsinki, 00014 Helsinki, Finland; § Natural Resources Institute Finland (Luke), 00790 Helsinki, Finland; ∥ Finnish Environment Institute, 00790 Helsinki, Finland; ⊥ The Sea, Environmental and Marine Biology, Åbo Akademi University, 20520 Turku, Finland; # Organismal and Evolutionary Biology Research Programme, Faculty of Biological and Environmental Sciences, University of Helsinki, 00014 Helsinki, Finland

**Keywords:** Bayesian networks, causal maps, ecological risk assessment, expert elicitation, multiple pressures, probabilistic modeling, seabed mining

## Abstract

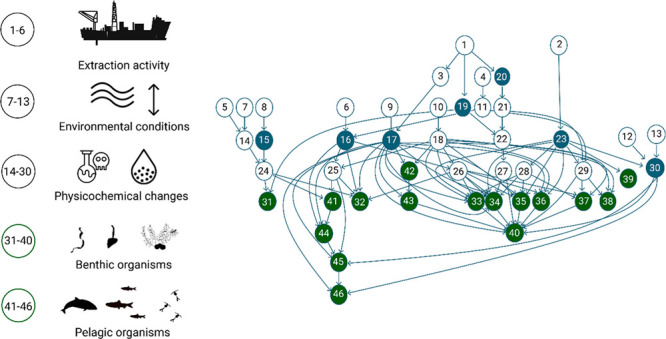

Mineral deposits containing commercially exploitable metals are of interest for seabed mineral extraction in both the deep sea and shallow sea areas. However, the development of seafloor mining is underpinned by high uncertainties on the implementation of the activities and their consequences for the environment. To avoid unbridled expansion of maritime activities, the environmental risks of new types of activities should be carefully evaluated prior to permitting them, yet observational data on the impacts is mostly missing. Here, we examine the environmental risks of seabed mining using a causal, probabilistic network approach. Drawing on a series of expert interviews, we outline the cause-effect pathways related to seabed mining activities to inform quantitative risk assessments. The approach consists of (1) iterative model building with experts to identify the causal connections between seabed mining activities and the affected ecosystem components and (2) quantitative probabilistic modeling. We demonstrate the approach in the Baltic Sea, where seabed mining been has tested and the ecosystem is well studied. The model is used to provide estimates of mortality of benthic fauna under alternative mining scenarios, offering a quantitative means to highlight the uncertainties around the impacts of mining. We further outline requirements for operationalizing quantitative risk assessments in data-poor cases, highlighting the importance of a predictive approach to risk identification. The model can be used to support permitting processes by providing a more comprehensive description of the potential environmental impacts of seabed resource use, allowing iterative updating of the model as new information becomes available.

## Abstract 2

This study examines how predictive risk assessment can inform marine resource governance and support environmental management plans for seabed mining.

## 1 Introduction

The increasing global demand for rare earth elements and other metals^1,2^ is driving interest in extracting minerals from the seafloor. Seabed mining activities are targeting different kinds of mineral ores and deposits^3^ found both within and outside national waters and exclusive economic zones, spanning a variety of environmental conditions and regulatory contexts. While most exploration concerns mining the deep seabed,^4^ the high cost and technological challenges of operating in the deep sea are driving further interest in mineral extraction from shelf seas.^5^ To avoid unbridled development of maritime activities, the impacts of new types of activities should be carefully evaluated prior to permitting them.^6^ However, dealing with impacts of activities that have not yet taken place means that there is no observational data on the impacts, with high uncertainties on both the implementation of the activity and its consequences for the environment. This uncertainty creates a challenge to estimate the impacts in a way that is scientifically robust, while accounting for the knowledge gaps to support decision-making.

Seabed mining will likely affect all levels of marine ecosystems, including the water column and the seafloor.^4,7,8^ The potential environmental impacts of mining have been addressed in an increasing number of studies,^9−12^ drawing on field studies, laboratory experiments, and associated modeling exercises. Even with valuable data from these studies, the impact experiments conducted to date offer a scattered view of the environmental impacts of mining. It is further uncertain to what extent the empirical disturbance studies succeed in scaling up to industrial mining operations.^10^

Environmental risk assessment (ERA) is a process aiming to evaluate the different possible outcomes following human activities.^13^ A risk in this context is defined as any unwanted event (here “impact”) and its probability. Currently, most ERAs build on estimating ecosystem responses to pressures based on vulnerability of the environment through semiquantitative scoring instead of the activity itself^14−16^ and as such are not well suited for describing different possible combinations of outcomes from new untested activities. By assuming additive relationships of pressures, these approaches often neglect the synergistic and antagonistic effects of pressures.^17^ A broader appreciation of the risks in the context of new maritime activities thus calls for improved systems thinking and integration of knowledge from multiple sources.^18^

Drawing on the recognition of causes and effects, causal chains or networks offer a systematic method to study environmental impacts.^19^ Although impact assessments are based on the concept of cause and effect, the use of explicit causal modeling has been little used in ERAs.^19−21^ Causal networks enable evaluating multiple scenarios and the underlying mechanisms in the studied system^22^ and have consequently been shown to be useful in policy interventions and management.^23,24^

Bayesian networks (BNs) are graphical models that represent a joint probability distribution over a set of variables and provide an alternative to commonly used scoring procedures in ERAs.^21,25^ In BNs, the strength of each connection between variables is described through conditional probabilities. As probabilistic models, the result of a BN is not a single point estimate but a probability distribution over the possible values of each variable, allowing estimating not only the most likely outcome but also the uncertainty associated with the estimates.^26,27^ BNs can thus synthesize outcomes of multiple scenarios by evaluating possible combinations of events and weighting them according to how likely they are. Given their modular structure, BNs can be used to support integrative modeling and can accommodate inputs from multiple sources, including simulations, empirical data, and expert knowledge.^28,29^ These properties make BNs well-suited for data-poor cases.^28^

Here, we describe an approach for integrating expert knowledge into a causal risk assessment for seabed mining. The approach builds on qualitative interviews, which are developed into a combined causal map through semiquantitative aggregation to build a quantitative risk model. We use the model to illustrate the impacts of mining in the Baltic Sea based on expert knowledge, as mining iron–manganese nodules has already been tested in an industrial setting in this area,^30^ and the ecosystem components and food web structure are well studied.^31−33^ Given the number of ongoing seabed mining initiatives and attempts to quantify impacts, the aim of this work is to provide a framework that allows both qualitative and quantitative information from multiple sources to be combined while explicitly addressing uncertainty. We further discuss how to operationalize quantitative risk assessments to inform decision-making, highlighting the importance of accounting for uncertainty in the context of emerging maritime activities.

## 2 Methods

**Figure 1:**
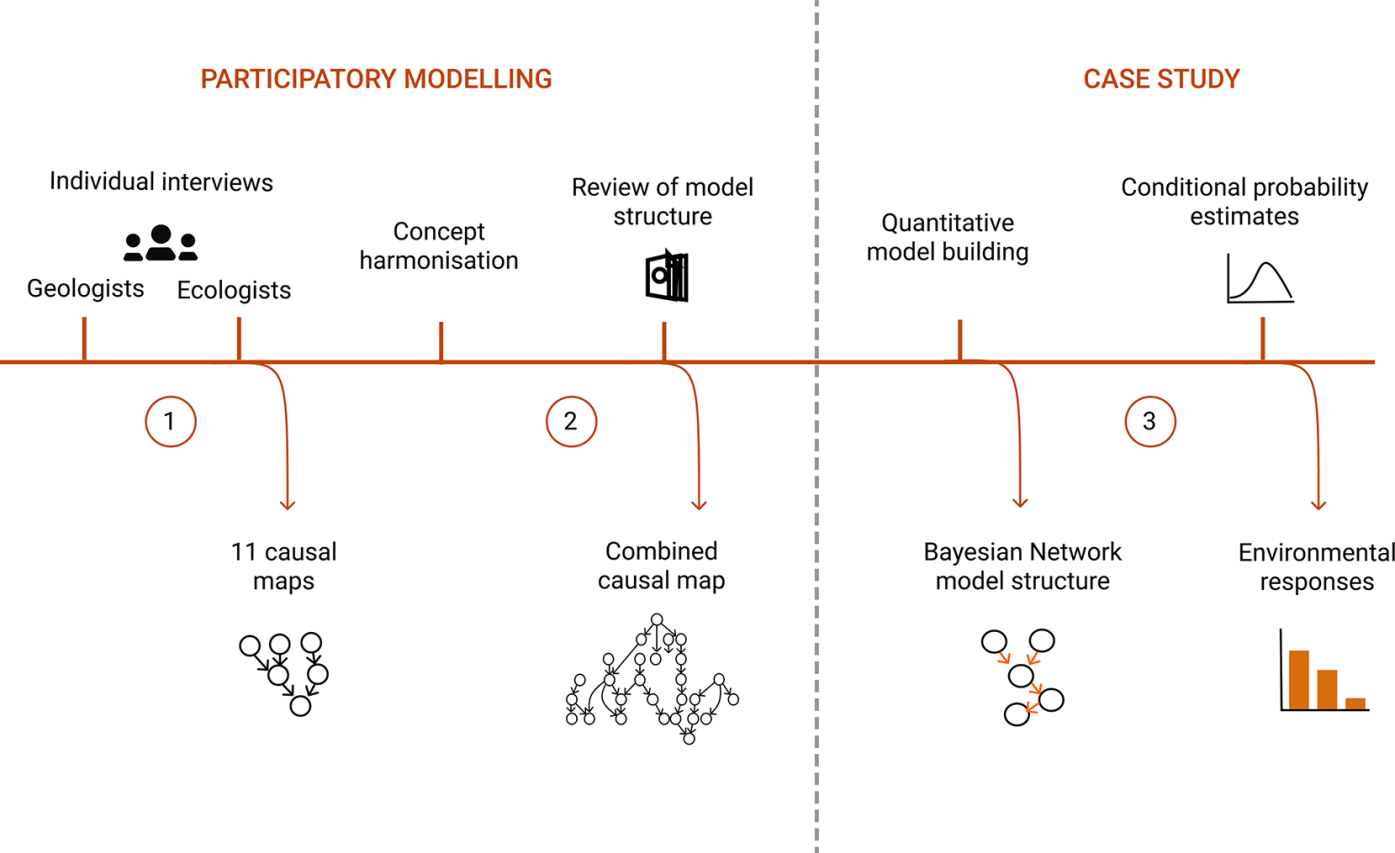
Conceptual figure of the modeling process summarizing the activities within the proposed approach (upper panel) and four main outcomes (lower panel). The approach builds on qualitative interviews (step 1) which are developed into a combined causal map through semiquantitative aggregation (step 2) to build a quantitative risk model (step 3).

We apply a three-step approach for working together with experts to create a model that summarizes the causal connections in the system and enables providing quantitative risk and uncertainty estimates (Figure 1). The first step consists of mapping the relationships between key drivers and ecosystem responses with experts in semistructured interviews. The use of structured methods for expert elicitation has been highlighted in recent years, and here, we follow a modified version of the IDEA (Investigate-Discuss-Estimate-Aggregate) protocol that consists of both individual and aggregated assessments from experts.^34,35^ Although the method is designed for quantitative estimates, we use it for qualitative causal mapping to test a structured approach for comprehensive interviews. In the second step, a combined model structure is created and reviewed by the experts in an iterative manner until a satisfactory model structure is obtained. The final step consists of quantifying the magnitude of the ecosystem impacts through conditional probabilities. A detailed description of the methods is given in the Supporting Information.

### 2.1 Case Study Background

Our case study deals with ferromanganese (FeMn) concretion removal in the northern Baltic Sea. The Baltic Sea is characterized by low species richness compared to many marine areas, and the food web structure and ecological traits characterizing major taxa have been well described.^36^ Due to the relatively shallow depth of the Baltic Sea, the extraction activity is to some extent comparable to sand and gravel extraction and would likely be performed by suction hopper dredging.^30^

**Figure 2:**
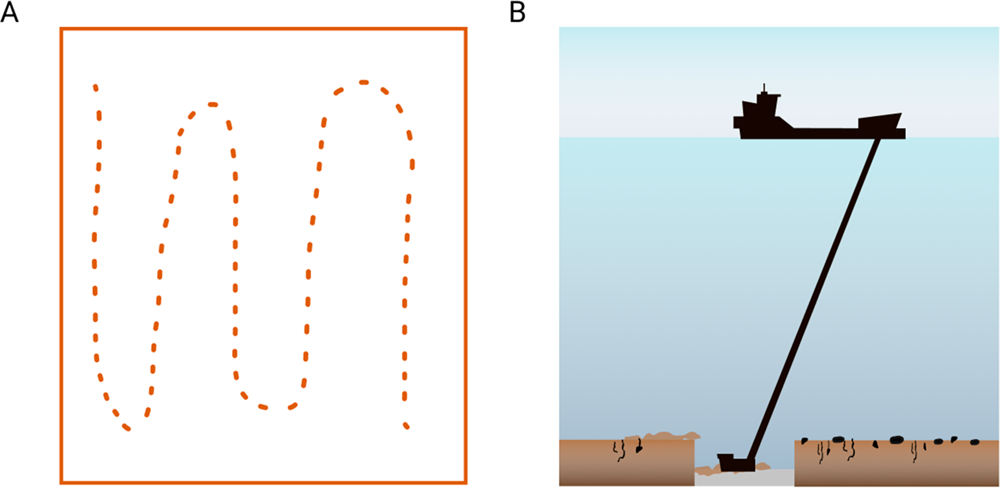
A) Plan view and B) profile view of mining a 1 km^2^ mining block. The dotted lines in panel A illustrate the extraction pattern of the mining device in a discrete block with FeMn concretions.

In our study scenario, mineral extraction is restricted to areas with a minimum depth of 40 m, assuming regulatory limits of such activities below the aphotic zone.^37^ The densest occurrences of FeMn concretions in Baltic Sea are also found below these depths.^38^ We assume that extraction is performed in a zigzag pattern in a limited extraction area of 1 km^2^, and it removes all concretions in the path of the suction head (Figure 2). Here, we assume homogeneous impacts on the areas that are not subject to direct extraction, although in reality the spatial footprint of impacts is dependent on the particle movement and distance of a point from the extraction area.^39,40^ Risks related to operating the vessels and impacts during transportation are not considered, as they are well addressed in other studies.^41^

### 2.2 Step 1: Expert Interviews

Framing the system and the connections between variables was performed as a causal mapping exercise with a multidisciplinary group of experts. The aim of causal mapping is to explore an individual’s view on a system under different scenarios by detailing the causes and effects. In an ERA context, this step constitutes the risk identification stage.^42^ Experts were recruited through snowball sampling by consulting researchers in different fields of marine sciences. To attain a diverse sample, we sent invitations to experts representing varying backgrounds in different institutes. Elicitation was performed gradually, which allowed us to evaluate when a sufficient number of experts had been interviewed by monitoring when the number of variables no longer increased with the addition of new experts. The final list of experts participating in the study included 11 experts from universities in Finland and Sweden, governmental research institutes, and intergovernmental organizations working on the Baltic Sea (see the SI for details on the experts).

The causal mapping exercise was conducted through semistructured interviews. We used individual interviews, as group interviews can be dominated by a small number of individuals,^43^ and experts’ judgments can be influenced by their peers.^44^ Interviews were held at a location chosen by the interviewee or online. For face-to-face interviews, causal maps were drawn on paper, whereas in online interviews, maps were constructed using an online drawing tool. All interviews were recorded with consent from the interviewee.

At the beginning of each interview, experts were introduced to the use of causal networks. Each expert was presented with the same scenario of the mining activity and the changes in the environment arising from the activity, denoted as pressures^45,46^ (Table 1). Details on how mining would likely be carried out and the resulting pressures were identified through a literature review^8^ and informal consultation with experts in geology and mineral resource extraction.

**Table 1:** Physicochemical Changes in the Environment (Pressures) Arising from Mining Used as a Starting Point in Causal Mapping with Experts

Pressure type	Description and references
Nodule removal	FeMn concretion removal from a mining block^30^
	contributes to loss of hard substrate on otherwise soft seabed
Modification of seafloor substrate type	Measure of changes in the sediment environment, including changes in
	-grain size^47^
	-sediment porosity^48^
	-sediment compaction^49^
	-organic enrichment^48,50^
	-pore water composition^51^
	-oxygen penetration depth^48,52^
Modification of seafloor topography	Changes in seafloor topography following extraction activities impacts^30,53^
Sediment dispersal in the water column	Total suspended solids concentration near the surface or in the water column both from the processing return and mining tool operation^40^
Sediment dispersal near seafloor	Total suspended solids concentration near the seafloor resulting from the processing return and mining tool operation^54^
Release of nutrients from the sediment	Release of soluble nutrients from the sediment plume to the seabed water column^55,56^
Release of toxic substances from the sediment	Release of contaminants from the sediment plume to the water column^57−59^
Underwater noise	Noise from the mining operation, including extraction of the substrate and vessel operations^60,61^

The first three interviews were held with marine geologists with experience in underwater mining technology. These interviews were used to adjust the pressures identified in a literature review^8^ and to identify environmental parameters and operational factors likely to affect the magnitude of the physiochemical changes arising from mining (Table 1). These variables form the core of the model by describing the basic processes related to mining.

To explore the ecological impacts arising from these pressures, the following eight interviews were conducted with marine ecologists. Each expert was presented with the same scenario of the mining activity and the physicochemical pressures identified in the first phase with the geologists (Table 1). The experts were then asked which ecosystem components they think will be affected by these pressures. Whenever possible, experts were asked to rate the strength of the causal connection on a 1–3 scale (3 being strongest). As the number of individual species even in the relatively species-poor Baltic Sea is too high to include in one model, we reduced this complexity by asking experts to address the affected organisms through the functional traits that would differentiate the effects on these organisms.

Experts were given unlimited time to complete the causal map and were informed that they may modify the causal map after the interview. After each interview (approximately 2–3 h each), the causal maps were digitized, and the resulting maps were sent to the experts for verification.

### 2.3 Step 2: Combining Causal Maps

To obtain a comprehensive view of the impacts of mining, the individual causal maps were combined into one causal network. To do this, we coded the connections between variables in the individual causal maps to adjacency matrices using the assigned link strengths whenever available. Prior to combining the maps, variables were harmonized and combined so that similar concepts were grouped under one variable. For instance, the terms “polychaetes”, “annelids”, and “worms” were grouped under “mobile infauna” (see the SI for individual maps).

The functional groups used in the assessment were compiled from the taxa and groups mentioned in the interviews and the trait expressions that were mentioned to affect the sensitivity to the pressures caused by mineral extraction.^60,61^ Most detail was given to the different groups of benthic fauna, and mobility, feeding mode, and position in sediment were used to group these organisms into broader groups (see the SI). The groups were set based on the expected response of organisms to the pressures caused by mining so that the traits characterize differential responses in the organisms (e.g., mobility increases an organism’s capacity to escape the mining area). Here, traits are treated as discrete variables, although most species express a variety of trait expressions.^62^

While elicitation of individual causal maps has been explored in-depth in the literature,^63,64^ there is little guidance on how to systematically combine diverse variables into one consensus map. In this work, all nonredundant variables and connections were included in the combined network. To ensure that the combined map represented the views of the experts involved in the model framing, experts had the opportunity to comment on the network structure in an open online document presented both in the form of a graph and a table. At this stage, the document and the comments were visible to all experts.

### 2.4 Step 3: Bayesian Network Model Development

The final causal network was used to develop a Bayesian network (BN) to provide quantitative estimates of the ecological consequences of mining under different mining scenarios. We quantified a submodel of the complete causal network focusing on three groups of benthic fauna: sessile filter feeding epifauna, mobile epifauna, and burrowing infauna. These groups were chosen for the demonstration as benthic fauna will be directly affected by mining activities, and these three groups were deemed to respond differently to pressures from mining in the expert interviews. The BN model was developed from variables describing these benthic faunal groups, the pressures affecting them, and any intermediate variables in the combined causal network. To reduce model complexity, we restricted the model to account only for the acute impacts through mortality within a spatially discrete mining block (Figure 2). To evaluate the model structure, we conducted a point-by-point appraisal of the causal connections in the model with three experts in marine ecology and geology with previous experience in seabed disturbance who had not participated in the model building.

Discrete variable states were defined based on literature and expert views (see Table 2). Variable states were set to reflect a reasonable variation in the variable, keeping the number of states to a minimum to facilitate further quantification. For improved application to other study areas, we use relative descriptions of pressures with relation to ambient conditions (e.g., low-high). To ensure that variable states are adequately set in terms of the study problem, discretization should be evaluated case-by-case based on both the availability of information and the scope of the assessment.^28,65^

**Table 2:** Variables in the Bayesian Network Model for Ecological Risks of Seabed Mining^a^

Variable category	Variable name	Description	Variable type	Possible states
Environmental conditions	Sediment type	Underlying sediment type	Random variable	Soft–hard-rocks^38^
	Contaminants in sediment	Concentration of toxic substances in the sediment	Random variable	Low-medium-high^G^
Extraction technique	Depth of extracted sediment	Depth of extracted sediment	Decision variable	<10 cm/11–30 cm/>30 cm^G^
	Volume of extraction	Volume of extracted sediment	Random variable	Low-medium-high^G^
	Processing return technique	Depth of the processing return of the excess sediment material	Decision variable	At the surface/at the bottom^71^
	Mining intensity	Proportion of concretions removed from the mining area	Decision variable	50%-75–100% removed^G^
Environmental changes	Suspended sediment	Suspended sediment near the seafloor	Random variable	Low-medium-high^E^^,^^G^
	Contaminant release	Release of toxic substances	Random variable	Low-significant^E^^,^^G^
	Sediment deposition	Amount of sediment deposited on the seafloor	Random variable	Low-medium-high^E^^G^
Affected functional groups	Sessile epifauna	Relative mortality of sessile epifauna	Random variable	0–10/11–30/31–60/61–80/81–100%^E^
	Infauna	Relative mortality of mobile infauna	Random variable	0–10/11–30/31–60/61–80/81–100%^E^
	Mobile epifauna	Relative mortality of mobile epifauna (fast-moving)	Random variable	0–10/11–30/31–60/61–80/81–100%^E^

a Random variables refer to variables with an associated probability distribution, whereas decision variables describe processes controlled by the party responsible for the extraction activity. References are given to variable states drawn from the literature, and expert informed states are denoted by G (geologist) or E (ecologist).

To quantify the magnitude of impacts between the pressures and the benthic faunal groups, we modeled the BN as an expert system, meaning that no empirical data is directly incorporated in the model. As direct elicitation of probabilities is a very labor intensive task,^66,67^ we used the graphical interface provided by the open source Application for Conditional probability Elicitation (ACE)^68^ to initialize the conditional probability tables (CPTs) with one expert in geology and one benthic ecologist. The application provides a starting point for defining the overall shape of a conditional probability distribution, which is done by ranking the direction and magnitude of the parent nodes on the child node and populating the table through a scoring algorithm.^68^

To assess probabilities of the impacts of direct pressures on benthic fauna, the CPTs initialized with the ACE application were evaluated and adjusted in a second session with another benthic ecologist. The total mortality of benthic fauna within a discrete block and one moment in time comprises the direct mortality from extraction of sediment and mineral concretions and the indirect mortality of the remaining fauna that are exposed to the pressures from the extraction activity. The probability of total mortality of benthic fauna was thus calculated as
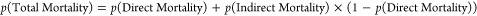
where *p*(Indirect Mortality) × (1–*p*(Direct Mortality)) accounts for the probability of the proportion of fauna remaining after direct extraction. In filling the CPTs, direct mortality was estimated to be directly proportionate to the mined area, and more detail was given to estimating the effects of the other pressures (see the SI for details). We applied numerical approximation at 1% accuracy to calculate joint probabilities of the combined discrete classes (Table 2) for total mortality used in the model.

The resulting CPTs were incorporated in the BN model (Figure 4) created in R software.^69^ The modeling was done using R 3.6.3, with package *bnlearn*.^70^ Full details of the model with the R scripts and the conditional probability tables are available at https://github.com/lkaikkonen/Causal_SBM.

BNs enable evaluating different scenarios and to compute posterior probabilities given new knowledge. In this context, a BN allows modification of the operational parameters to evaluate the impacts of different mining operations and the associated changes in the functional groups. The joint probability distribution in the BN may then be used to make queries on the impact of multiple pressures on specific ecosystem components to assess the risks and to evaluate which variables should be monitored to obtain a reasonable overview of the impacts. For demonstration, we queried the network on two alternative mining scenarios defined with experts, which we define as a combination of specific states of the decision variables that describe the overall mining process and are assumed to be controlled by the party responsible for the mining operation (Table 2). The random variables in the model are further affected by these decision nodes (Figure 4, Table 2).

## 3 Results

### 3.1 Causal Maps

The expert interviews resulted in 11 individual causal maps. In some cases, the experts took the lead in drawing the variables and connections between them, whereas in most interviews, the modeler had the main responsibility of drafting the map based on the discussion.

The number of variables in the individual maps varied between 8 and 24. There were no contradictory views between experts regarding the direction of the causal connections in the system, and the differences between the maps were attributed to the number of variables and level of detail in different processes regarding the impacts of mining. We were not successful in eliciting all link strengths, and only the strongest connections were explicitly given by all experts. The individual causal maps are included in the Supporting Information (S1).

**Figure 3:**
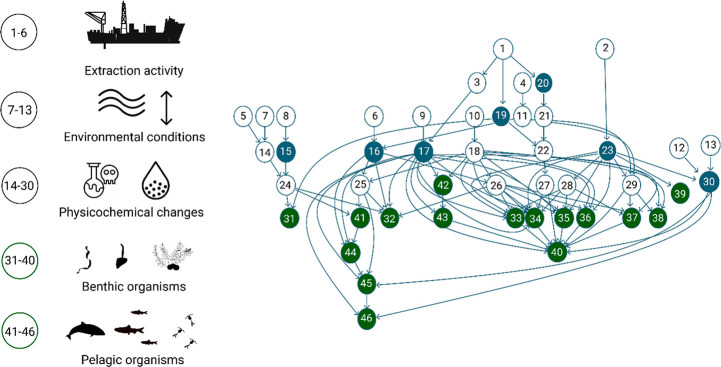
Simplified representation of the combined causal map of the environmental impacts of FeMn concretion extraction on Baltic Sea ecosystem. The numbers refer to the number of variables under each variable category. The blue circles denote the pressures that were used as a starting point for the causal mapping, and green circles denote biological variables. For full details of the variables and causal connections, see Tables S4–S6 and Figure S7 in the Supporting Information.

After concept harmonization, the final causal map has 53 variables. Multiple iterations of expert comments on the causal network structure resulted in a combined causal network with 96 connections (Figure 3). The rationale for the connections between variables and further details on them are summarized in Tables S4–S6 in the Supporting Information.

### 3.2 Impacts of Mining on Marine Ecosystems: Combined Causal Network

The first set of interviews with geologists revealed several factors affecting the magnitude of physicochemical changes in the environment, related to both the mining operation and the prevailing environmental conditions (Table 2). The factors regarding the mining technique included water depth at the extraction site, depth of extracted sediment, and processing return technique. Both the geologists and ecologists included several environmental factors in their causal maps, including variables describing the sediment characteristics and composition, water column chemistry, and hydrological parameters (Figure 3).

The impacts on the biological ecosystem components were more complex in terms of the spatial and temporal dimensions than the physicochemical changes. Experts successfully adopted a parsimonious attitude to defining the functional groups and expressed how these groups would be affected by the different pressures. The most detail in terms of functional traits was given to benthic fauna which are most directly affected by substrate extraction. Experts included a wide range of organisms in the assessment that were unlikely directly affected in the extraction area, including early life-stages of fishes, macrophytes, and mammals. Factors affecting the recovery potential of organisms and ecosystem functions after disturbance were mentioned in all interviews.

Direct extraction of seabed substrate and the resulting habitat loss was deemed to have the most significant impact on benthic fauna. Many experts equally considered the impacts of elevated suspended sediment concentrations on filter feeding organisms severe. In the interviews, the functional groups were deemed different in terms of acute impacts of disturbance. For example, while highly mobile organisms like fish are assumed to escape from the extraction area, significant changes in the environment either through modification of bottom substrate or benthic fauna as food are expected to potentially affect the distribution of demersal fish species. Similarly, release of contaminants from the sediment was estimated to significantly affect all organisms, yet it was noted that many toxic effects might only be expressed in the reproductive success of organisms. Nearly all experts noted the negative impacts of underwater noise on mammals and fishes.

### 3.3 Quantitative Case Study: Acute Impacts on Benthic Fauna

**Figure 4:**
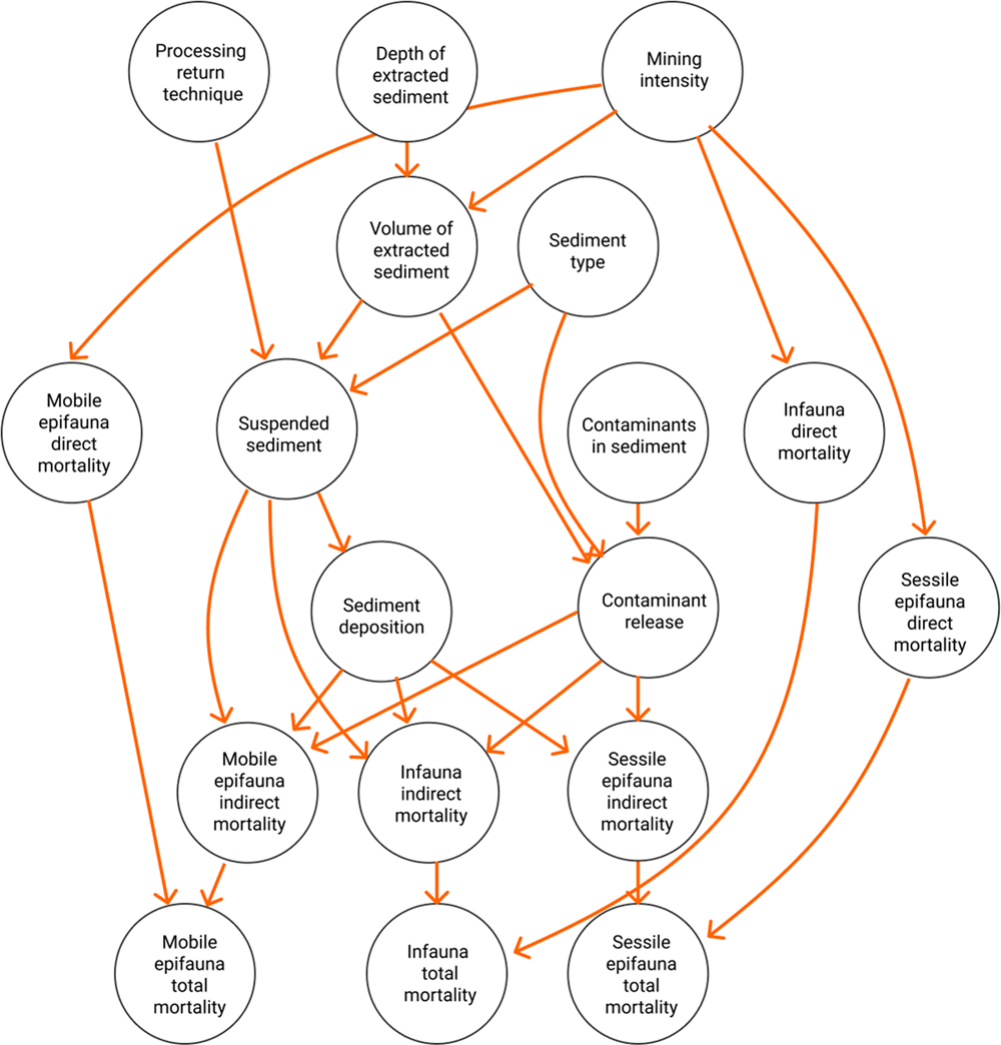
Bayesian network structure for immediate impacts on selected groups of benthic fauna. Mining scenario may be controlled by *processing return technique*, *depth of extracted sediment*, and *mining intensity*.

**Figure 5:**
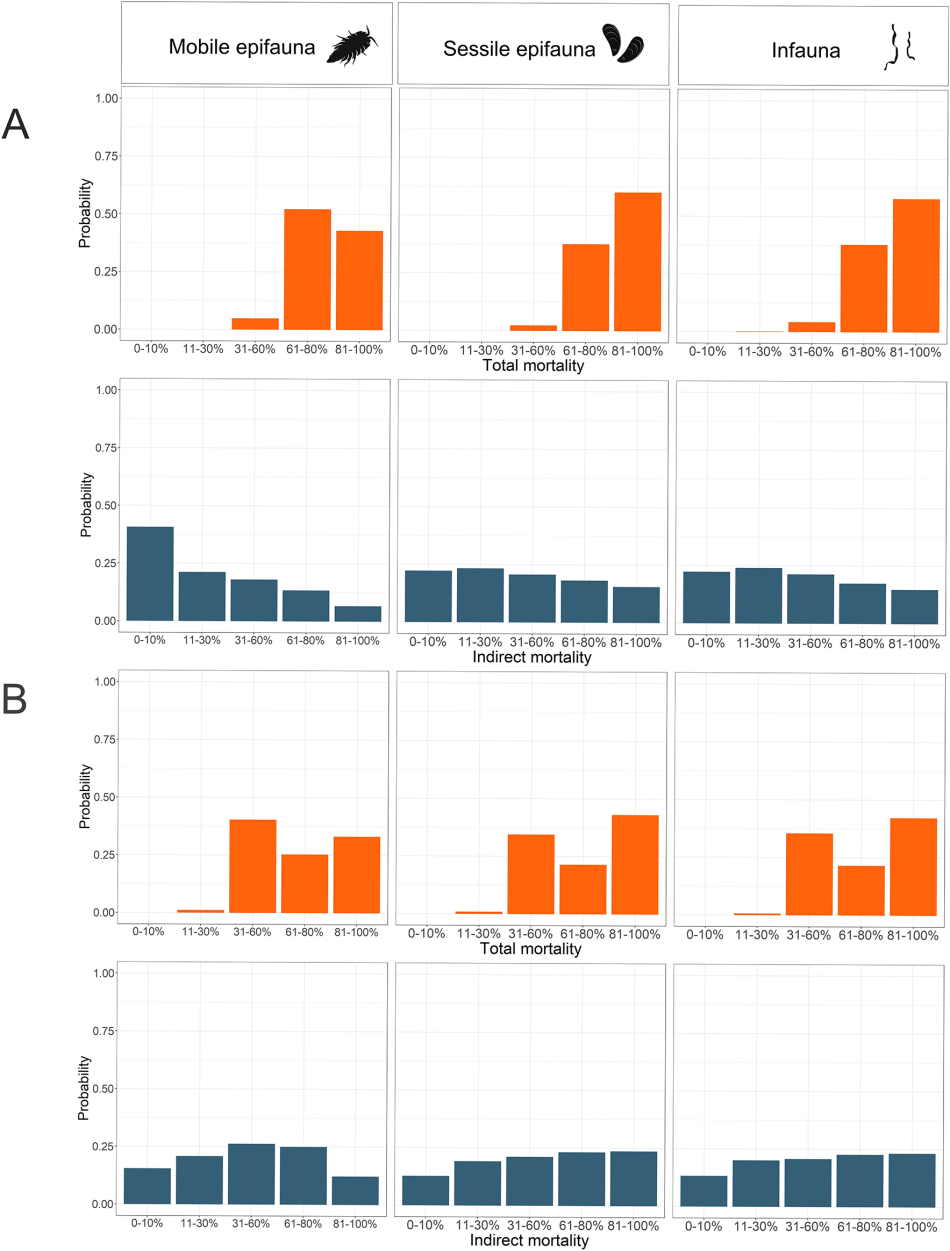
Joint probability distribution of the total and indirect mortality of mobile epifauna, sessile epifauna, and infauna under two alternative mining scenarios: A) mining 75% of a discrete mining block with 11–30 cm sediment extracted and B) mining 50% of a discrete mining block with 11–30 cm sediment extracted with release of harmful substances from the sediment. Orange bars depict result on total mortality, and blue bars depict result on indirect mortality of fauna.

The full causal model is highly complex (Figure 3), and parameter estimation would be a demanding task. Therefore, for illustration, we selected 18 variables for the quantitative analysis to describe the acute impacts on benthic fauna (Figure 4,Table 2). We queried the network on two different mining scenarios. The resulting probability distributions are presented in Figure 5.

In the case of mining 75% of a discrete mining block, the most probable outcome in terms of total mortality for both sessile epifauna and infauna is estimated to be 81–100% mortality (Figure 5A). The probability of the highest mortality for sessile epifauna is slightly higher than for infauna (60.1% compared to 57.7%, respectively). For mobile epifauna, 60–80% mortality is the most likely outcome with a 52.2% probability.

The likeliest outcome of the mining scenario described above in terms of indirect mortality resulted in indirect mortality of 11–30% of both infauna (24.1% probability) and sessile epifauna (23.3% probability) and 0–10% mortality of mobile epifauna with 40.7% probability (Figure 5A). The probability of the highest mortality (81–100%) is 14.8% for infauna, 15.5% for sessile epifauna, and 6.6% for mobile epifauna. Overall, the probability of both indirect and direct mortality on sessile epifauna and infauna are deemed equally widely distributed.

The BN model allows estimating the probability of any variable of interest in the model (here relative mortality) given certain evidence (e.g., regarding the mining operation or environmental conditions). To give an example, when mining occurs on only 50% of a discrete block, but release of harmful substances is known to occur, the probabilities for the indirect mortality of benthic fauna are higher for all groups (Figure 5B). These changes illustrate the relative importance of certain pressures on the overall mortality.

Changes in the extent of direct extraction of seabed substrate and FeMn concretions had the largest impact on the direct mortality of the benthic fauna. In terms of indirect effects, the release of ecologically significant levels of toxic substances from the sediment had the highest impact on the mortality of benthic fauna. In a similar way, the model may be used to evaluate the cumulative effects of multiple stressors for each assessed ecosystem component by first ranking the relative effects of each stressor on the mortality of the community and then evaluating the probability distribution for each combination of stressor levels.

## 4 Discussion

This study evaluates the ecological risks of seabed mining using a causal probabilistic approach. By interviewing a multidisciplinary group of experts, we outline a basis for an ecological risk assessment model. We further demonstrate how qualitative information may be used to move toward a quantitative assessment to estimate the impacts of seabed mining on benthic fauna in the Baltic Sea. These results show that the knowledge related to the impacts of seabed mining even in a well-known system is still low, calling for further research on the risks of mining if the operation permits are to be based on a valid scientific understanding.

### 4.1 Expert Knowledge in Ecological Risk Assessments

Involving multiple experts in consecutive interviews provided a comprehensive view of the pressures arising from mining and the affected ecosystem components. Particularly the interviews with geologists enabled the inclusion of operational variables related to the mining activity and the environmental conditions governing the magnitude of pressures. While we had expected experts to prioritize their own fields’ species in more detail, the experts’ previous participation in similar mapping exercises seemed to be main the factor governing the number of connections and variables. For this reason, the optimal number of experts to comprehensively evaluate the system in question may vary significantly and should be evaluated for each case study.

Although many of the impact pathways described in the obtained causal maps have been identified in previous studies,^72,73^ our mapping exercise enabled a more detailed inclusion of pelagic ecosystem components which have been neglected in many previous studies.^72,74−76^ A qualitative causal representation of the impacts alone can thus help better understand how risks emerge and can potentially be mitigated.^24,77^ Drafting the causal maps with experts from the beginning further ensures that all relevant connections are included, and biases in thinking will be revealed easier.^78,79^

Overall, the probability distributions on the relative mortality of benthic fauna from expert assessment show low levels of certainty on the impacts. One reason for this is likely the lack of scientific knowledge, particularly regarding the cumulative effects from multiple pressures,^80^ which make validating such assessments challenging.^81,82^ Although the different groups of benthic fauna were deemed to experience differential responses from sediment deposition and suspended sediment, the probability distributions describing these effects are very similar between infauna and sessile epifauna. While these results may be a consequence of the high uncertainties related to the impacts, further knowledge engineering approaches to facilitate elicitation^43,83^ could offer insights into the effects of multiple pressures. Future development of the model should thus address improving the quantitative estimates of the risks in terms of both methodology and the used evidence.

The interviews and the subsequent causal mapping highlighted the challenges in conceptualizing spatiotemporal complexity related to anthropogenic impacts.^84^ Although we specifically requested experts to focus on a discrete spatially defined area and immediate impacts, factors affecting recovery and spatial extent of impacts arose in all interviews. These differences in temporal scale are a result of changes in the environment varying in their scope and persistence (Table S8), resulting in immediate impacts, chronic and long-term impacts, and factors affecting the recovery potential of organisms.

Given these challenges, attempting direct modeling of such dynamic systems may not be appropriate, as it can result in excessive simplification and loss of information. Giving the experts free hands was beneficial for capturing the nonimmediate impacts, and in retrospect, our interviews could have been developed in a more flexible manner. We posit, however, that providing starting points for the assessment by setting the spatial and temporal limits helped the experts to get started without being tangled in the multidimensionality. The results show that it is essential to consider effects from multiple perspectives and account for the multidimensional disturbance space. An operational assessment should thus include multiple time steps or account for continuous effects and changes in the prevailing conditions.

### 4.2 How Can Predictive Risk Assessment Inform Marine Resource Governance?

The paucity of evidence on the impacts of seabed mining calls for more comprehensive views of the risks and knowledge gaps to support decision-making.^85^ With recent calls for more empirical approaches to broad scale seabed mining initiatives,^86^ new data on the impacts of mining may be incorporated in the risk model to learn the probability distributions between the nodes from data and further be completed with expert assessment. Such models thus offer a framework to synthesize empirical findings to support operational risk assessments.

Given the modular structure of BNs, the model presented here may be adapted for more complex ERAs through searate layers and submodels. For instance, accounting for the indirect mortality separately allows further refining the assessment to account for the impacts of indirect effects, as these are deemed significant in terms of the spatial footprint due to sediment dispersal.^87,88^ While this model provides only a limited view of the ecosystem, it is a starting point for more detailed ERAs and may be complemented by different ecological, spatial, and temporal dimensions,^89^ including recovery of ecosystems^90^ and foodweb interactions.^91^

Another advantage of probabilistic approaches is that the conditional probabilities may be drawn from multiple sources and can include both qualitative and quantitative data. This allows iterative updating of the model as new information becomes available, for instance by incorporating data on the ecological consequences of specific pressures to organisms from laboratory experiments.^92^ Although little data would be available, such as in the case of most deep-sea ecosystems,^93^ BNs are ideal in data-poor cases,^28^ and the paucity of knowledge will be explicitly reflected in the probability estimates. Similarly, expressing where information is lacking through expert interviews is equally valuable^94^ and supports the application of the precautionary approach. As a next step, this approach could be applied to a region with empirical data on the impacts of mining as a means to synthesize available information complemented by expert knowledge.

To support decision-making on potential future use of seabed resources, model simulations under alternative mining scenarios should be compared to existing policy targets regarding acceptable changes in ecosystems. Using a quantitative approach offers a more robust and transparent means of estimating the impacts of emerging activities when defining acceptable thresholds to the impacts.^95^ Estimating the impacts and accounting for the knowledge gaps with a probabilistic approach can aid to either support a moratorium and not to go ahead with exploitation in line with a precautionary approach^96^ or to provide information for more comprehensive risk management plans for potential future mining activities, including the need for mitigation measures.^97^ In cases where uncertainties are considered too high, permits could be made to be conditional on improved knowledge by allowing only one mining operation to proceed until impacts have been documented in more detail,^98^ urging the industry to carry out further studies.

Although the risks of offshore activities are most often approached through environmental impacts, there are many economic and societal considerations to be accounted for.^99−101^ Causal networks may be enhanced into more comprehensive frameworks for integrated environmental assessments to promote integration of diverse values and stakeholder views.^102,103^ Engaging with multiple sources of knowledge not only strengthens the knowledge base for assessing the risks but also allows revealing possibly contradictory views between experts and stakeholders^104,105^ to support better outcomes for both the marine environment and society.^106^

The expanding industrial use of the ocean space and resources calls for more detailed assessments on the risks associated with them. Recent incentives for more sustainable marine governance^106−108^ further urge applying an ecosystem approach to resource management, including impact and risk assessments of activities on both the marine ecosystem and human society. Based on the results of this study, we posit that while empirical observations are key in unraveling the impacts of novel activities, full consideration of the different scales of risks requires a systematic approach to integrate findings from empirical studies, modeling, and expert assessments. An improved view of the risks as an underlying concept in research on the impacts of seabed mining will aid developing integrative ecosystem based management of emerging maritime industries.^109^

## Supporting Information Available

S1, detailed description of methods, Table S2, information on experts involved in causal mapping exercise; Figures S3.1–S.11, complete causal maps from expert interviews; Tables S4–S6, causal interactions and causal connections, Figure S7, combined causal network, and Table S8, spatiotemporal extent of stressors (PDF)

## Notes

Full details of the model with the R scripts and the conditional probability tables for this article may be accessed at https://github.com/lkaikkonen/Causal_SBM.
