# Supplementary material for: Causal Approach to Determining the Environmental Risks of Seabed Mining
Source: Environ Sci Technol. 2021 Jun 21;55(13):8502–13. doi: 10.1021/acs.est.1c01241 (PMC8277135; doi:10.1021/acs.est.1c01241)
Supplement: Supplementary file 1 — es1c01241_si_001.pdf [file es1c01241_si_001.pdf]

Supporting information for:

A causal approach to determining the environmental risks of seabed mining

Laura Kaikkonen[1,2], Inari Helle[2,3,6], Kirsi Kostamo[4], Sakari Kuikka[1], Anna Törnroos[5], Henrik Nygård[4], Riikka Venesjärvi[3], Laura Uusitalo[4]*

[1] Ecosystems and Environment Research Programme, Faculty of Biological and Environmental Sciences, University of Helsinki, 00014, Helsinki, Finland

[2]Helsinki Institute of Sustainability Science (HELSUS), University of Helsinki, 00014, Helsinki, Finland

[3] Natural Resources Institute Finland (Luke), 00790, Helsinki, Finland

[4] Finnish Environment Institute, 00790, Helsinki, Finland

[5] Åbo Akademi University, Environmental and Marine Biology, The Sea, 20520 Turku, Finland

[6]Organismal and Evolutionary Biology Research Programme, Faculty of Biological and Environmental Sciences, 00014, University of Helsinki, Finland

*Corresponding author: Laura Kaikkonen, Ecosystems and Environment Research Programme, Faculty of Biological and Environmental Sciences, University of Helsinki, Finland, email: laura.kaikkonen@iki.fi

This pdf includes:

S1. Detailed description of the methods.

Table S2. Information on the experts involved in the causal mapping exercise.

Figures S3. Complete causal maps from the expert interviews.

Table S4 Causal interactions between ecosystem components and the pressures affecting them.

Table S5. Causal connections between the physicochemical pressures and other model variables.

Table S6. Causal connection between other model variables.

Figure S7. Combined causal network.

Table S8. Spatiotemporal extent of the stressors.

S1 *Methods description*

A causal approach to determining the environmental risks of seabed mining

Step 1: Causal mapping with experts to define model structure

The first stage of the work focuses on defining which ecosystem components should be included in the risk model and mapping out their interdependencies based on a previous network drawn from literature.

Model building consisted of first interviews with experts in geology and mining technology to recognize the pressures caused by mineral extraction and affected non-biological ecosystem components (e.g. changes in seabed topography, sediment composition), and then with experts in ecology to complete the pressures and the affected ecosystem components.

Each expert was presented the same scenario of FeMn concretion extraction (including e.g. water depth at extraction site and extraction technique), and was then asked which ecosystem components would likely be affected by the pressures resulting from extraction activities. This first round of interviews resulted in 11 individual causal maps detailing the pressures caused by mineral extraction, the affected ecosystem components, as well as a number of additional variables

We note that the sufficient number of experts is context-specific, and very different numbers of participating experts can be found from the literature (see e.g. the review by Krueger et al[1]). Clemen and Winkler[2] highlighted the diminishing marginal returns of large numbers of experts and suggested using 3–5 experts, whereas Morgan[3], for instance, emphasized that if the experts share similar views of the science underpinning their understanding of the system, 5–6 experts is enough, but a larger group is needed if the experts express diverse opinions. In our work, we included new experts in a stepwise process until no further information (e.g. new variables) was introduced to the model. Hence, we adopted the "theoretical saturation" concept which suggests continuing sampling until information is simply confirmed but not modified or elaborated[1].

While we believe that 11 experts formed an adequate sample in our study, we also recognize that this may not always be the case, especially when dealing with poorly known systems, where information on the different ecosystem components and processes may be very scattered to topic experts. However, it should be noted that the difference between poorly known and well-known systems is not straightforward, as the experts can disagree on relatively fundamental issues even in systems that are studied relatively thoroughly, like the Baltic Sea (see e.g. Uusitalo et al.[4]).

Step 2: Finalizing the model structure

The causal maps resulting from the first interviews were combined into a consensus map, which all participating experts had the possibility to comment on.

2.1 Adjacency matrices

The first step in constructing the combined causal map consisted of listing all different variables in the individual causal maps. We the evaluated which concepts described the same variable or

process (e.g. “oxygen concentration” and “hypoxia”) and removed any duplicate variables (see also section 2.2. on functional groups).

Using these same harmonized variable names for all the causal maps, we then coded the connections in each individual causal map into a combined adjacency matrix, which illustrates the connections between the pressures and the affected ecosystem components (see example in Table 1). To evaluate the views between experts, we summed the number of times a connection was mentioned in the interviews.

We had also asked the experts to rank the strength of the causal connection from 1-3 (3 being strongest), but due to the high number of connections in many of the maps, we were not able to elicit all the link strengths. For this reason, and for wishing to illustrate a complete picture of the different impact pathways, we ended up including all unique variables into the combined causal map. However, first summarizing the causal connections into tables using either average link strengths of the number of times the connection was mentioned in the interviews would be useful in cases where there is disagreement between experts as to the direction of the causal connection, or if there is a need to limit the number of connections in the model for e.g. further quantification of the model. In our case, we used the information on the link strengths when selecting the variables for the Bayesian Network model.

Table 1. Extract from an adjacency matrix used to combine the individual causal maps to one consensus map. The numbers denote the number of maps that included a specific connection.

**Pressure/Ecosystem component**	Underwater noise	Modification of seafloor substrate type	Concretion removal
Mammals	5	0	2
Demersal fish	5	2	2
Pelagic fish	5	0	1
Fish eggs	0	0	0
Fish larvae	1	0	0
Phytoplankton	0	0	0
Zooplankton	0	1	1
Pelagic crustacea	1	0	1

2.2 Functional groups

The final functional groups included into the model were combined of the taxa and functional groups directly mentioned in the interviews, and the trait expressions that were mentioned to define the vulnerability to the pressures caused by mineral extraction (e.g. feeding habit). An example of all the possible trait combinations for benthic fauna is presented below (Figure 1).

The number of trait expressions/modalities used to determine the groupings varied between functional groups. Although the theoretical number of random trait groupings can be potentially large, only a small subset of combinations are likely present and further considered in the combined network model. If a trait was not seen to affect the response in some broader group of organisms, all the different combinations of trait expressions were not used. Here, traits are treated as discrete variables for simplification, although in nature species may express a variety of traits. These

functional groups have been drafted based on the expected acute response. Any additional traits affecting recovery potential could be added in further model development.

Figure 1. Combinations of the full combination of trait expressions. Only those combinations of trait expressions observed in the Baltic Sea were considered in the combined network.

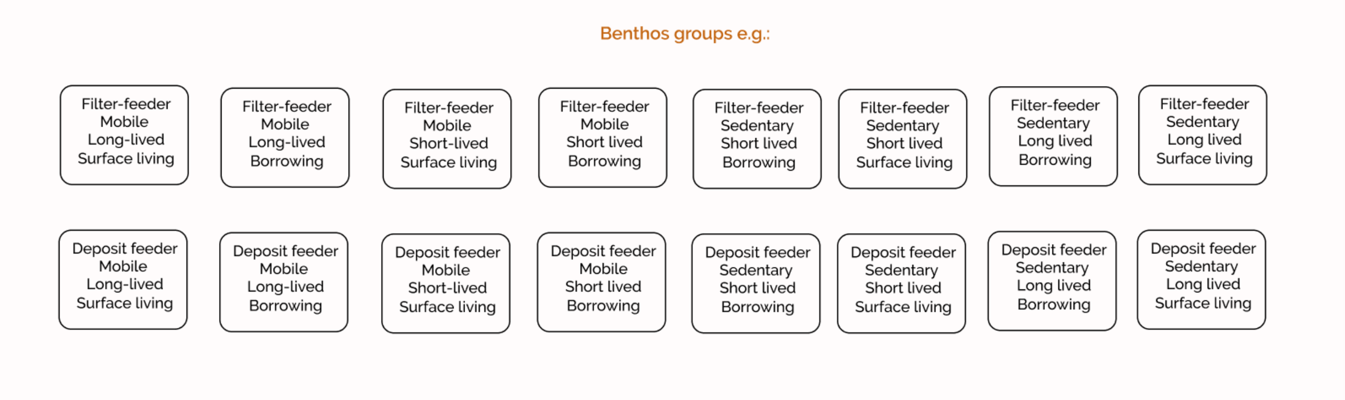

2.3 Review of model structure

To ensure that the combined map represented the views of the experts involved in the model framing, all participating experts had the possibility to comment on the model structure in an open online document. Given the large size of the model, the causal network was presented both in the form of a graph (Figure S7) and a table (Table S4-S6). The document and the comments on the model structure were visible to the other experts to encourage knowledge exchange and learning between participants. The experts were also given the option to send their comments directly to the modeller.

Step 3: Bayesian Network building

The final causal model was modelled as a Bayesian network (BN). Bayesian statistics provide an alternative to commonly used simple scoring procedures in ecological risk assessment. For an introduction to BNs, see e.g. [5–7]. The BN was modelled as expert system, meaning that no empirical data was directly incorporated in the model. The modelling was done using R statistical software, with package “*bnlearn*”.

Based on the results of the expert interviews, we quantified only a sub-model of the complete causal network focusing on three groups of benthic fauna: sessile filter feeding epifauna, mobile epifauna, and burrowing infauna. The BN model was developed from variables describing these three benthic faunal groups, the main pressures affecting them, and any intermediate variables between them in the combined causal network. To reduce complexity of the model in terms of spatial and temporal dimensions of the impacts in the first stage of the work, we restricted the model to account only for the acute impacts within a spatially discrete mining block.

3.1 Defining discrete variable states

Discrete variable states were defined to describe the variation in the magnitude of pressures arising from the mining activity. This included considering which combinations of pressures the extraction and sediment deposition are likely to result in. The discrete states may be described through quantitative metrics, like different concentrations of substances or depth in centimeters, or can be

based on qualitative descriptions of discrete classes, like high, medium, low. The key aspect with regard to the biological responses was that these pressure levels should make sense from an ecological perspective. As the demonstration in our case study is not bound to a specific setting, we decided to frame the model variables quite generally with the states low-moderate-high. The discrete variable states were mostly defined based on expert judgement, informed by literature.

3.2 Probability elicitation

Within a BN, the magnitudes of impacts are illustrated through conditional dependencies. The probabilities of each value of the child node, conditioned on every possible combination of values of the parent nodes, were drawn from expert opinion. These describe the strength of the causal relationships between variables in the model (Figure 2).

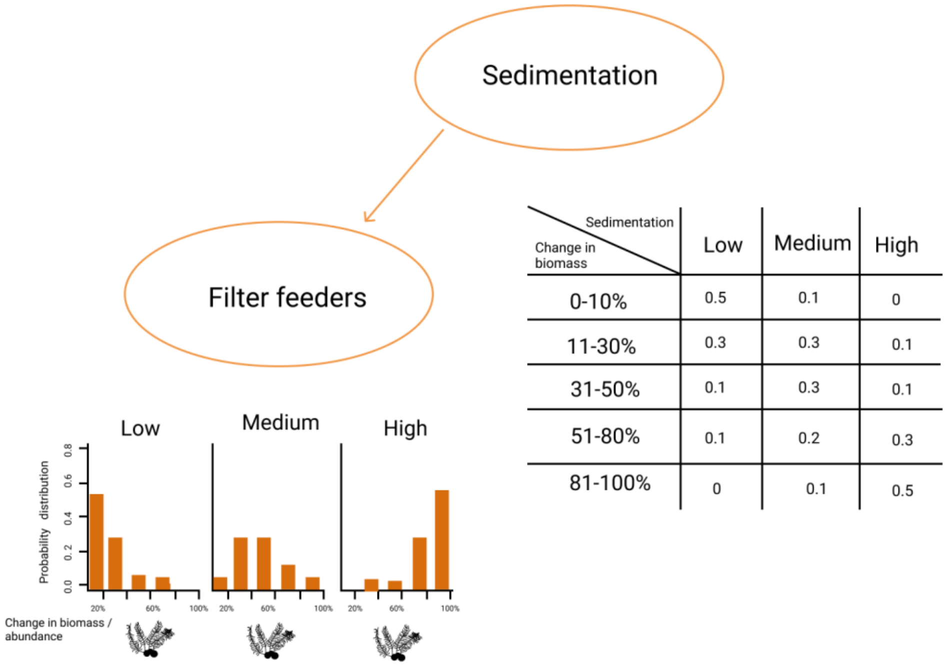

Change in biomass \ Sedimentation	Low	Medium	High
0-10%	0.5	0.1	0
11-30%	0.3	0.3	0.1
31-50%	0.1	0.3	0.1
51-80%	0.1	0.2	0.3
81-100%	0	0.1	0.5

Figure 2. Schematic description of the conditional probabilities within two nodes in a BN. In this example, the change in biomass of filter feeding organisms is conditionally dependent on the levels of sediment deposition. The conditional probability table (CPT) describes the values of both the parent and the child node, summarising the probability distribution of mortality of benthic fauna under different levels of sedimentation.

We used the graphical interface provided in the ACE application [8] to initialise the conditional probability tables (CPTs) with one expert in geology and one benthic ecologist. The application provides a starting point for defining the overall shape of a conditional probability distribution by allowing ranking the direction and magnitude of the parent nodes on the child node and populating the table through a scoring algorithm. For the probabilities concerning the impacts of direct

pressures on benthic fauna, the prefilled tables evaluated and adjusted in another session with another benthic ecologist to reach a consensus on the magnitude of the impacts.

Direct and indirect mortality were modelled separately, so that the total mortality of benthic fauna comprises both the direct mortality from the extraction of sediment and mineral concretions and the indirect mortality stemming from the other pressures from the extraction activity. This allows for estimating the effects of the pressures for both the direct mining area (total mortality) and in neighbouring areas (indirect mortality).

Direct mortality was estimated as a direct proportion of the mined area, so that e.g. mining 50% of an area results in 50% of fauna being extracted. For the remaining (indirect) pressures, we first populated the CPTs with one benthic ecologist using the ACE application and then refined them using Google sheets. To evaluate the first estimates, we organized another session with another benthic ecologist s who had been involved in the model building and was available for further work to finalize the CPTs on indirect pressures (Table 2). We elicited the frequencies using the following questions:

- What is the lowest the value could be?
- What is the highest the value could be?
- What is the most likely value?

Table 2. Example of a finalized conditional probability table (CPT). The CPT summarizes all possible combinations of the different variable states for a given node. The probabilities were elicited in frequencies and further converted into probabilities.

**Parent nodes (pressures)**			**Child node (target variable of interest)**				
**Suspended sediment**	**Sediment deposition**	**Contaminant release**	**0-10% mortality**	**11-31% mortality**	**31-60% mortality**	**61-80% mortality**	**81-100% mortality**
Low	Low (<1mm)	Low	79	15	5	1	0
Low	Low (<1mm)	Significant	29	27	20	18	6
Low	Moderate (1-5mm)	Low	69	20	7	3	1
Low	Moderate (1-5mm)	Significant	10	25	32	26	7
Low	High (5-10mm)	Low	10	28	40	17	5
Low	High (5-10mm)	Significant	7	15	35	33	10
Moderate	Low (<1mm)	Low	75	14	7	3	1
Moderate	Low (<1mm)	Significant	22	29	24	20	5
Moderate	Moderate (1-5mm)	Low	33	27	20	13	7
Moderate	Moderate (1-5mm)	Significant	10	17	39	27	7

Moderate	High (5-10mm)	Low	32	26	23	14	5
Moderate	High (5-10mm)	Significant	20	19	30	23	8
High	Low (<1mm)	Low	33	27	20	13	7
High	Low (<1mm)	Significant	10	17	39	27	7
High	Moderate (1-5mm)	Low	32	26	20	14	8
High	Moderate (1-5mm)	Significant	5	13	20	32	30
High	High (5-10mm)	Low	31	25	20	15	9
High	High (5-10mm)	Significant	5	13	19	32	31

Finally, the separate CPTs for both direct mortality and indirect mortality were combined so that the total mortality of benthic fauna within a discrete block and one moment in time comprises the direct mortality from extraction of sediment and mineral concretions, and the indirect mortality of the remaining fauna that are exposed to the pressures from the extraction activity (i.e. one organism cannot be both extracted and die of sediment deposition). The probability of total mortality of benthic fauna was thus calculated as:

$$P(Total\ mortality) = P(Direct\ Mortality) + P(Indirect\ Mortality)\ x\ (1 - P(Direct\ Mortality))$$

where p(Indirect Mortality) x (1-p(Direct Mortality)) accounts of the probability of the proportion of fauna remaining after direct extraction. While the resulting joint probabilities are continuous, here we calculate them at 1% accuracy (e.g. round them up) to provide the probabilities of a mortality of a given proportion of benthic fauna for the five discrete classes we use in our model.

References

(1) Krueger, T.; Page, T.; Hubacek, K.; Smith, L.; Hiscock, K. The Role of Expert Opinion in Environmental Modelling. *Environ. Model. Softw.* **2012**, *36*, 4–18. https://doi.org/10.1016/j.envsoft.2012.01.011.

(2) Clemen, R. T.; Winkler, R. L. Combining Probability Distributions from Experts in Risk Analysis. *Risk Anal.* **1999**, *19* (2), 187–203.

(3) Morgan, M. G. Use (and Abuse) of Expert Elicitation in Support of Decision Making for Public Policy. *Proc. Natl. Acad. Sci.* **2014**, *111* (20), 7176–7184. https://doi.org/10.1073/pnas.1319946111.

(4) Uusitalo, L.; Korpinen, S.; Andersen, J. H.; Niiranen, S.; Valanko, S.; Heiskanen, A.-S.; Dickey-Collas, M. Exploring Methods for Predicting Multiple Pressures on Ecosystem Recovery: A Case Study on Marine Eutrophication and Fisheries. *Cont. Shelf Res.* **2016**, *121*, 48–60. https://doi.org/10.1016/j.csr.2015.11.002.

(5) Charniak, E. Bayesian Networks without Tears. *AI Mag.* **1991**, *12* (4), 50–50.

(6) Jensen, F. V. *An Introduction to Bayesian Networks*; UCL press London, 1996; Vol. 210.

(7) Uusitalo, L. Advantages and Challenges of Bayesian Networks in Environmental Modelling. *Ecol. Model.* **2007**, *203* (3), 312–318. https://doi.org/10.1016/j.ecolmodel.2006.11.033.

(8) Hassall, K. L.; Dailey, G.; Zawadzka, J.; Milne, A. E.; Harris, J. A.; Corstanje, R.; Whitmore, A. P. Facilitating the Elicitation of Beliefs for Use in Bayesian Belief Modelling. *Environ. Model. Softw.* **2019**, *122*, 104539. https://doi.org/10.1016/j.envsoft.2019.104539.

Table S2. Experts involved in the causal mapping. All interviewed experts have a broad expertise of the Baltic Sea ecosystem and the specific fields of expertise are not comprehensive.

**Field/Specific expertise**	**Name**	**Institution**
Marine geology	Henry Vallius	Geological Survey of Finland
Marine geology	Aarno Kotilainen	Geological Survey of Finland
Marine biogeochemistry	Jouni Lehtoranta	Finnish Environment Institute
Benthic ecology/Trait-based approaches	Anna Törnroos	Åbo akademi Univeristy
Benthic ecology	Alf Norkko	University of Helsinki
Benthic ecology	Sebastian Valanko	International Council for the Exploration of the Sea
Benthic ecology	Henrik Nygård	Finnish Environment Institute
Ecosystem ecology/Macrophytes	Kirsi Kostamo	Finnish Environment Institute
Coastal fish	Meri Kallasvuo	Natural Resource Institute Finland
Ecosystem ecology/Food webs	Lena Bergström	Swedish University of Agricultural Sciences
Ecosystem ecology/Pelagic ecosystems	Ilppo Vuorinen	Archipelago Research Institute, University of Turku

S3 Complete causal maps from the expert interviews. The colored ovals depict the pressures which were presented at the beginning of the interviews and served as a starting point for the causal mapping exercise. Numbers from 1 to 3 indicate the strength of the causal connection, 3 being the highest.

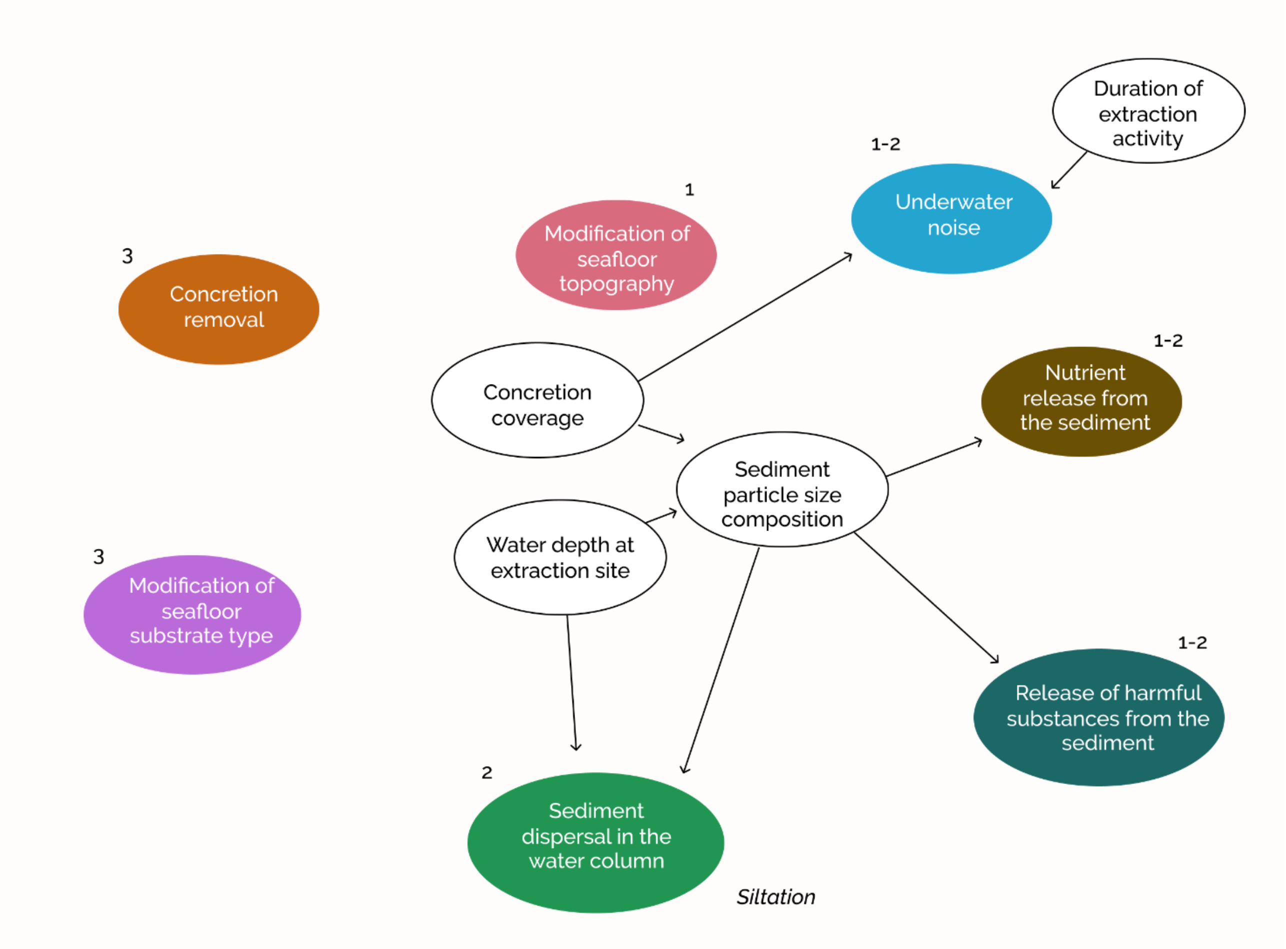

Figure S3.1 Interview 1 with a marine geologist. Numbers from 1 to 3 indicate the strength of the causal connection, 3 being the highest. The colored ovals depict the pressures which were presented at the beginning of the interviews and served as a starting point for the causal mapping exercise

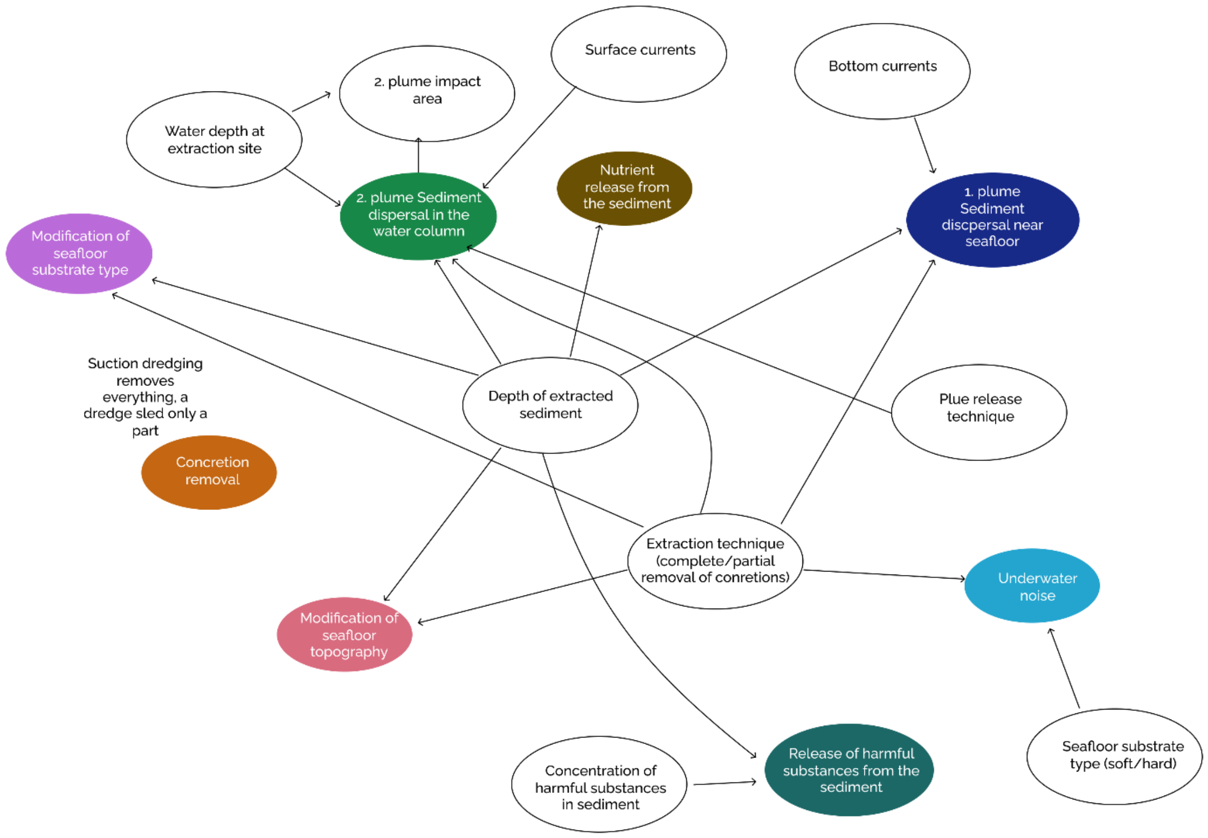

Figure S3.2 Interview 2 with a marine geologist. The colored ovals depict the pressures which were presented at the beginning of the interviews and served as a starting point for the causal mapping exercise.

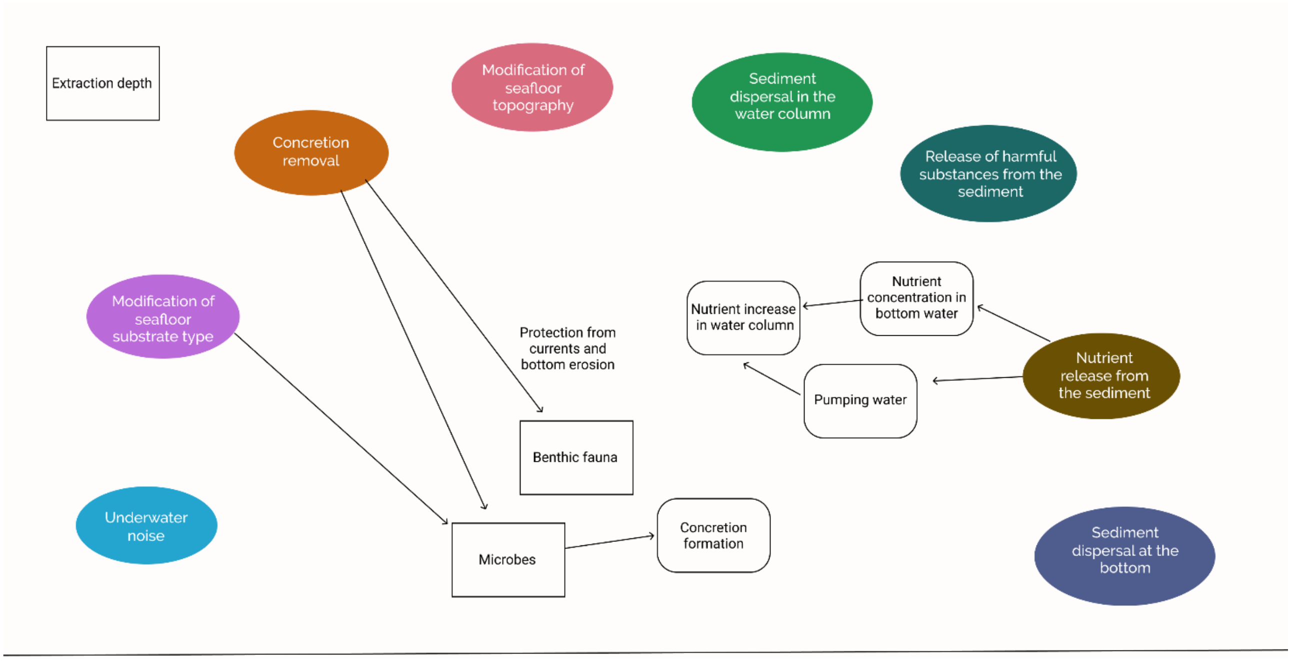

Figure S3.3 Interview 3 with a marine geologist. The colored ovals depict the pressures which were presented at the beginning of the interviews and served as a starting point for the causal mapping exercise.

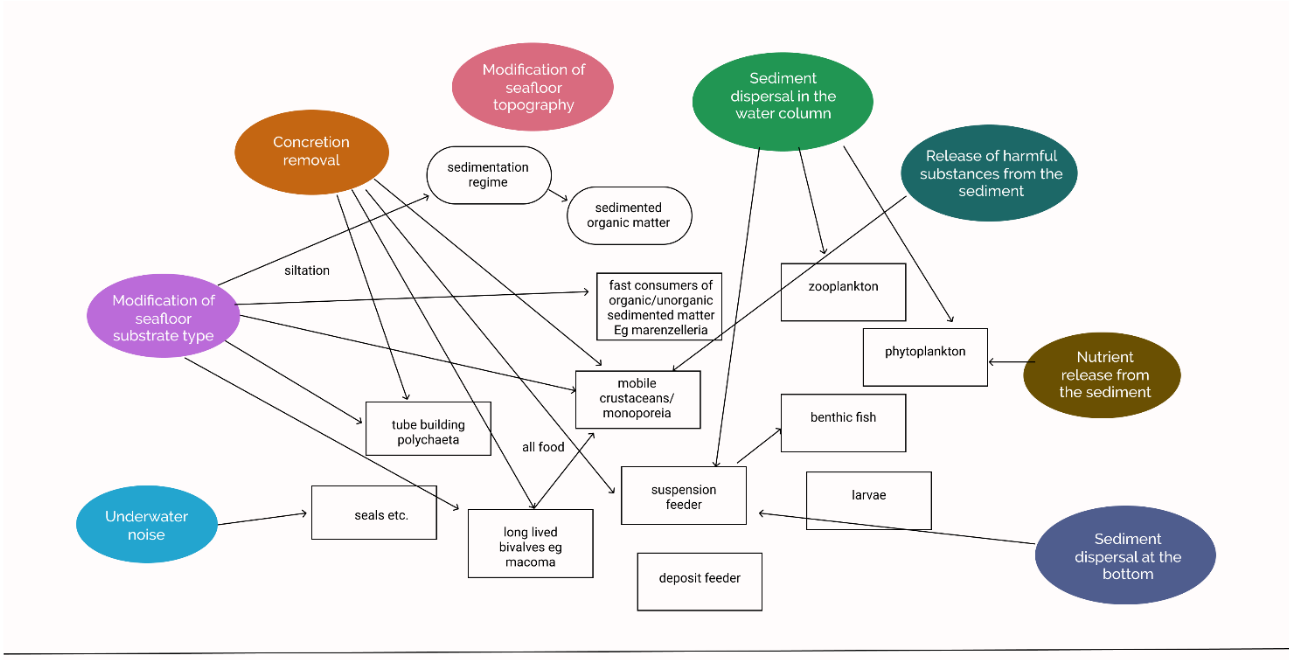

Figure S3.4 Interview 4 with a marine ecologist. The colored ovals depict the pressures which were presented at the beginning of the interviews and served as a starting point for the causal mapping exercise.

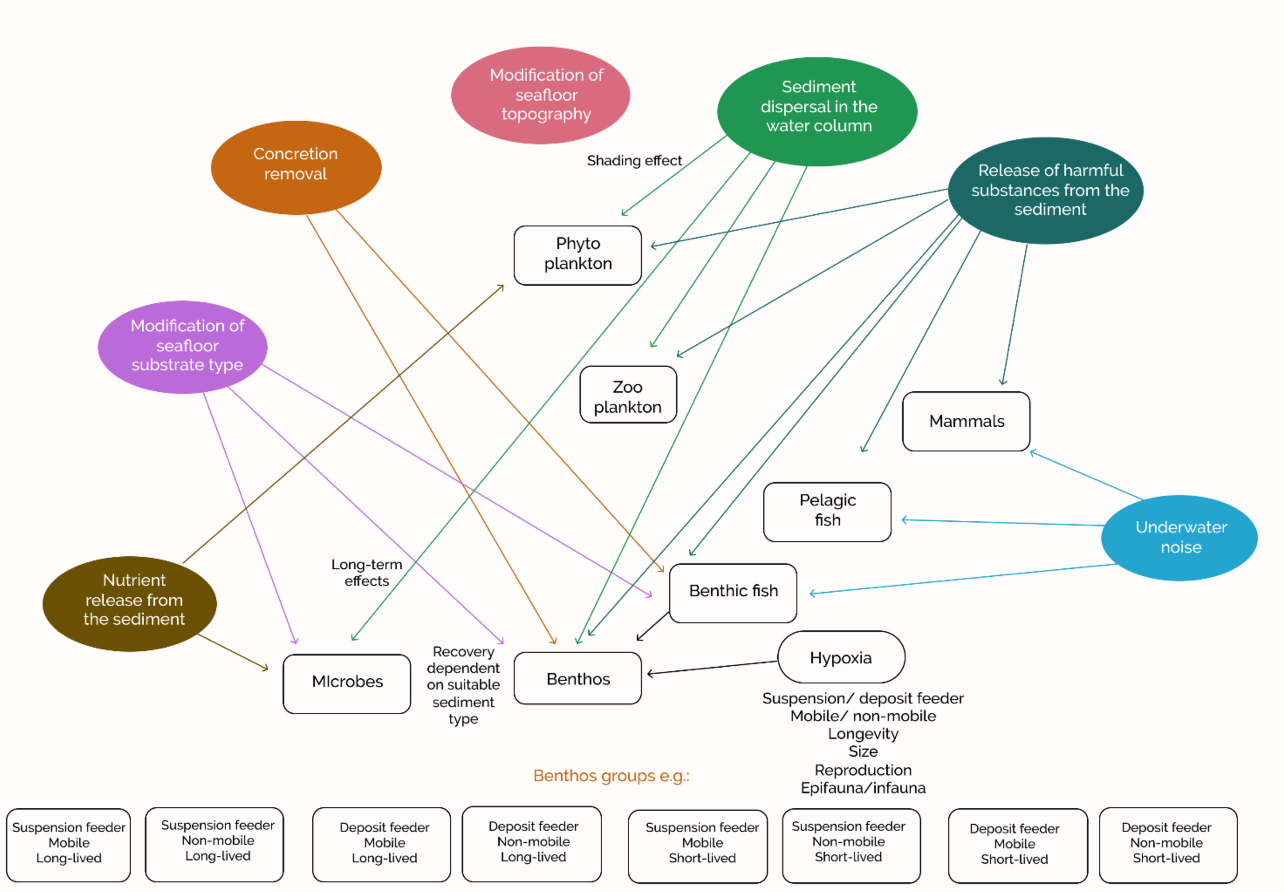

Figure S3.5 Interview 5 with a marine ecologist. The colored ovals depict the pressures which were presented at the beginning of the interviews and served as a starting point for the causal mapping exercise.

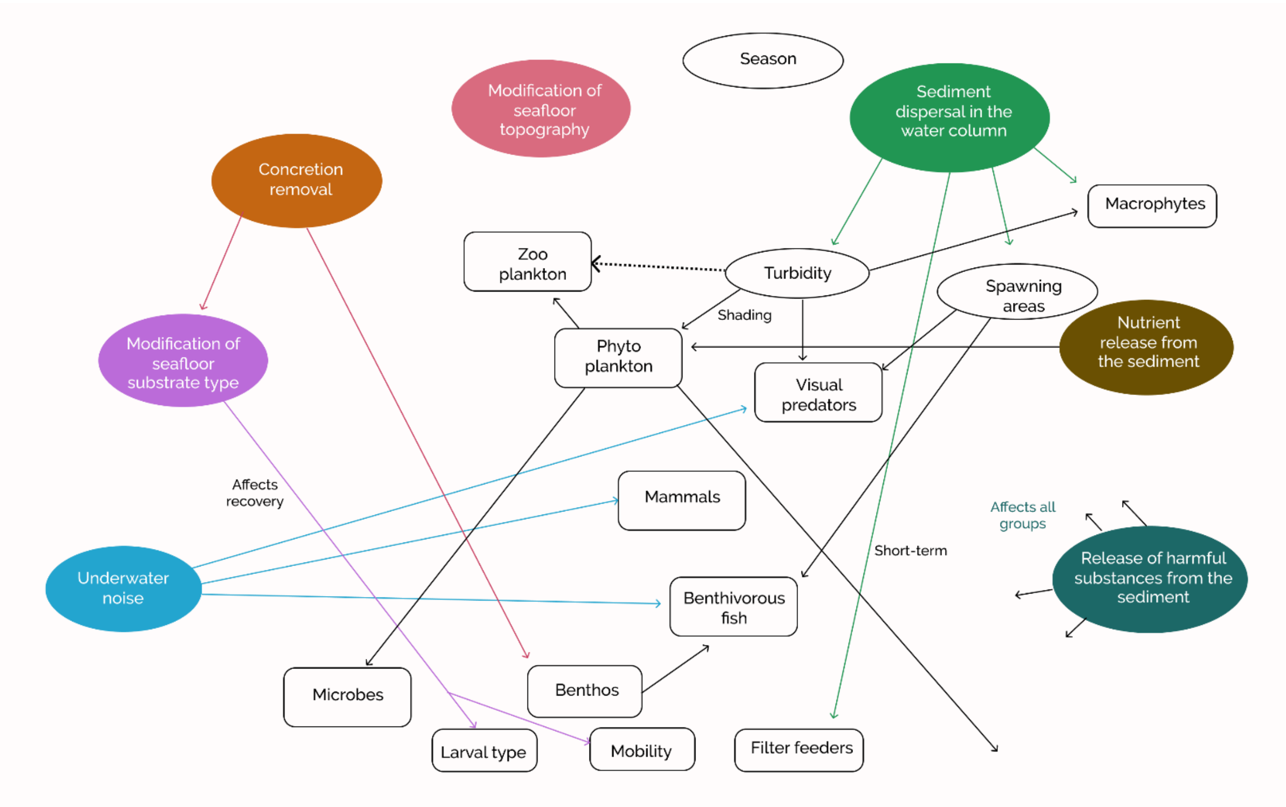

Figure S3.6 Interview 6 with a marine ecologist. The colored ovals depict the pressures which were presented at the beginning of the interviews and served as a starting point for the causal mapping exercise.

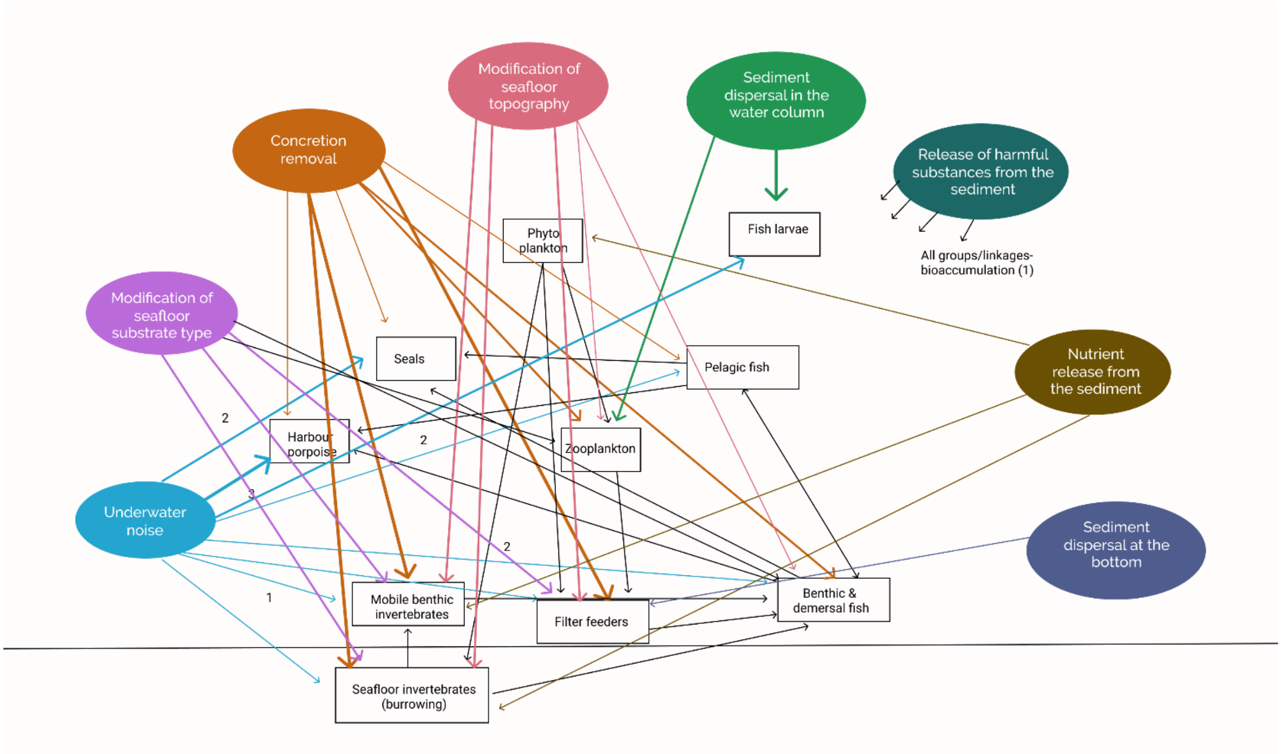

Figure S3.7 Interview 7 with a marine ecologist. The colored ovals depict the pressures which were presented at the beginning of the interviews and served as a starting point for the causal mapping exercise. Numbers from 1 to 3 indicate the strength of the causal connection, 3 being the highest.

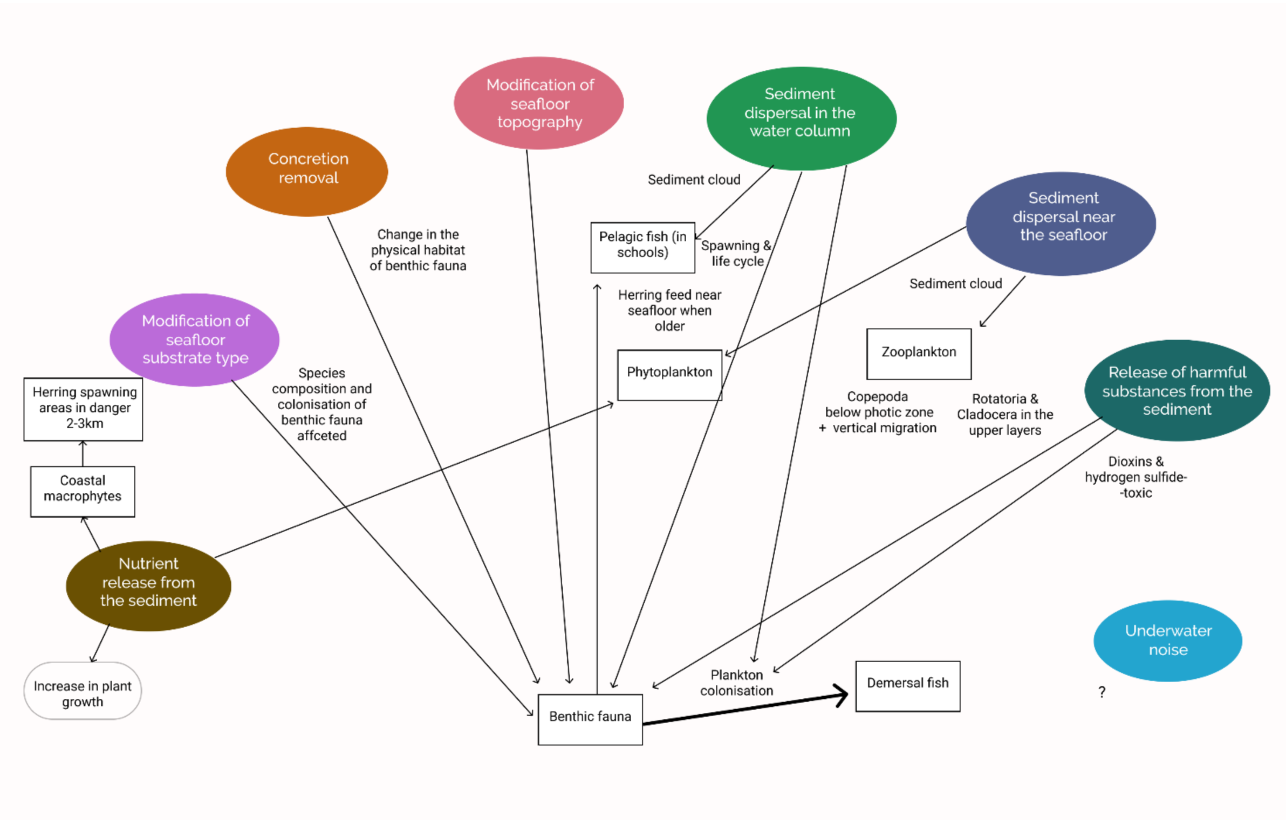

Figure S3.8 Interview 8 with a marine ecologist. The colored ovals depict the pressures which were presented at the beginning of the interviews and served as a starting point for the causal mapping exercise.

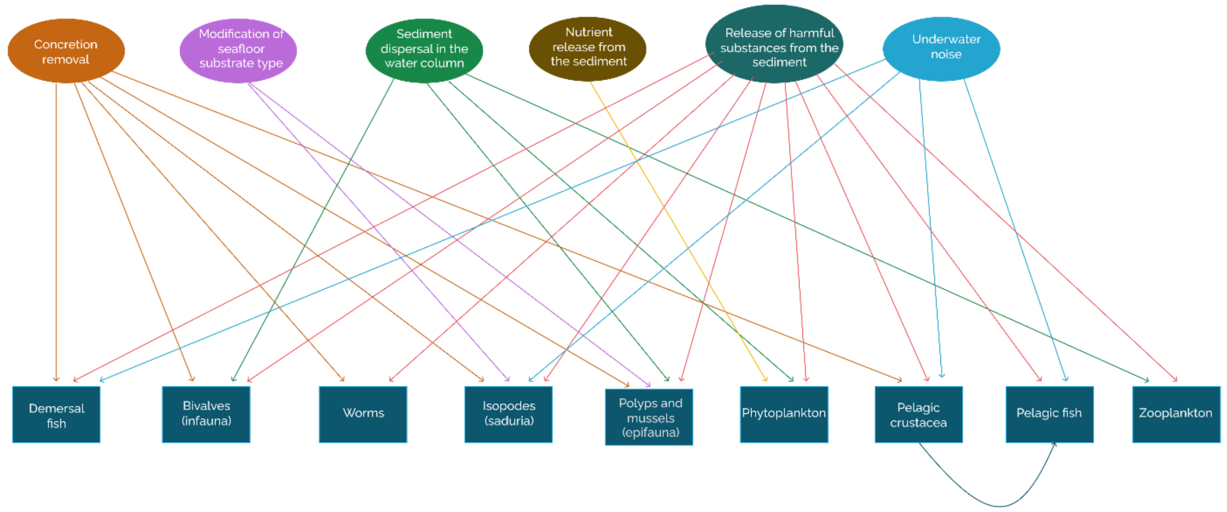

Figure S3.9 Interview 9 with a marine ecologist. The colored ovals depict the pressures which were presented at the beginning of the interviews and served as a starting point for the causal mapping exercise.

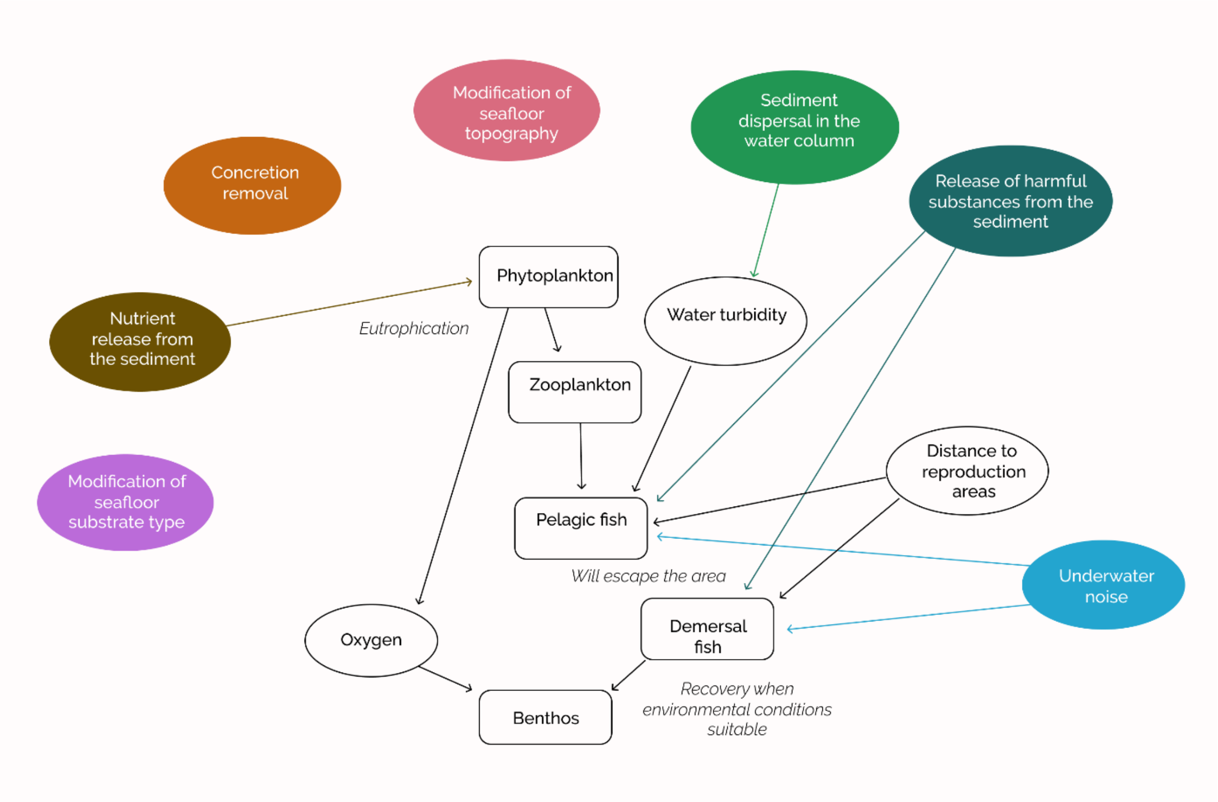

Figure S3.10 Interview 10 with a marine ecologist. The colored ovals depict the pressures which were presented at the beginning of the interviews and served as a starting point for the causal mapping exercise.

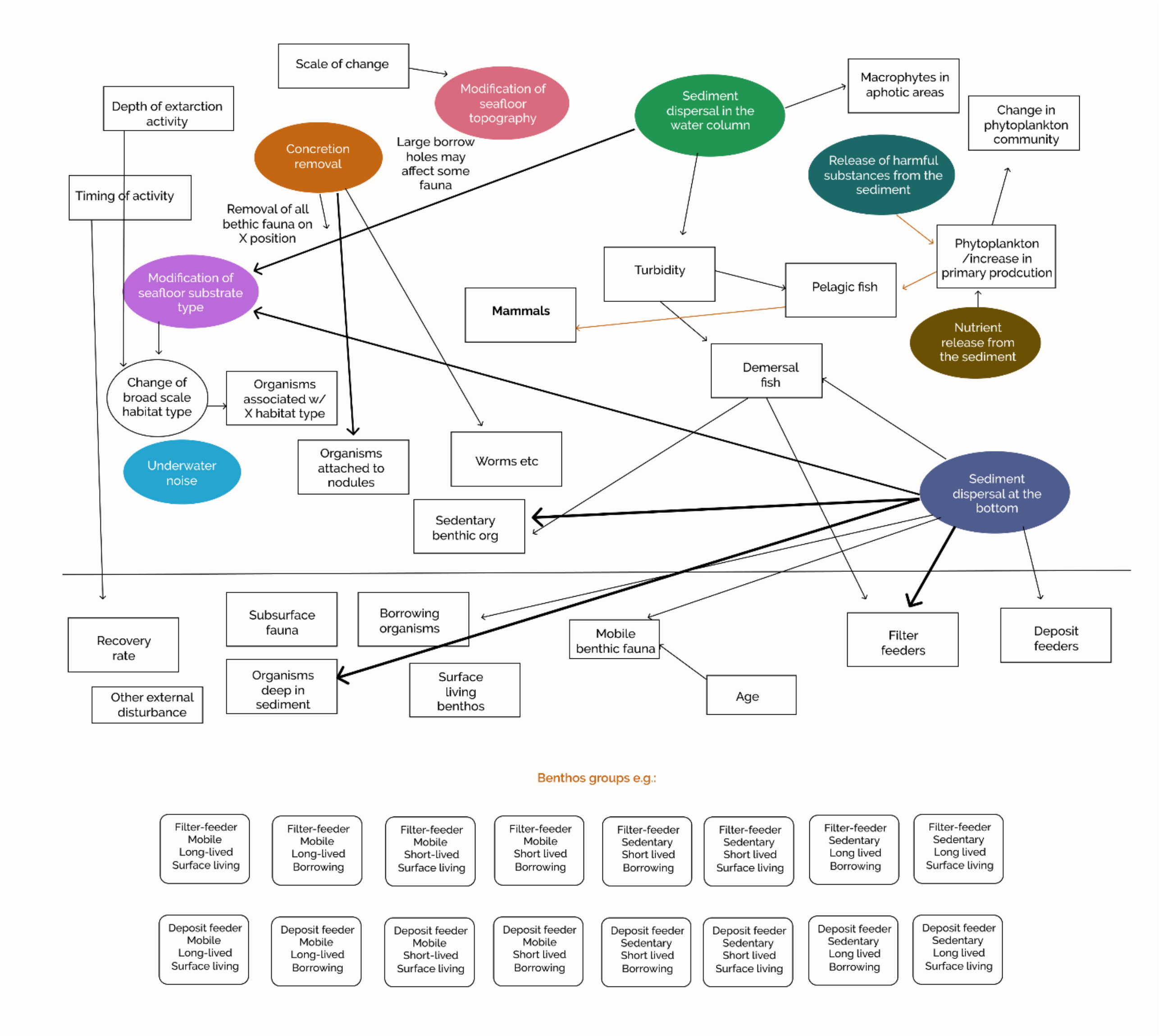

Figure S3.11 Interview 11 with a marine ecologist. The colored ovals depict the pressures which were presented at the beginning of the interviews and served as a starting point for the causal mapping exercise. The different arrow strengths indicate the strength of the causal connection (thickest being strongest).

Table S4. Causal interactions between ecosystem components & pressures affecting them.

Functional groups of organisms	Affecting pressures or other variables
**Mammals (porpoises & seals)**	**1.** Underwater noise induces stress.<br>**2.** Harmful substances may reduce fitness in mammals.<br>**3.** Quantity and quality of fish as food source affect the number of mammals in the area.
**Demersal fish**	**1.** Underwater noise induces stress & causes fish to migrate away. Migration requires energy, which reduces the fitness of individuals.<br>**2.** Sediment dispersal in the water column & on the seafloor may cause fish to avoid extraction area during activity.<br>**3.** Harmful substances reduce fitness in fish, which can be reflected in the reproductive success of individuals.<br>**4.** Change in habitat type may cause fish to leave area.<br>**5.** Anoxia causes fish to avoid extraction area.<br>**6.** Quantity of benthic invertebrates as food affects the number of demersal fish in the area.
**Pelagic fish**	**1.** Underwater noise induces stress & can cause fish to avoid extraction area.<br>**2.** Zooplankton biomass as food affects number of pelagic fish in the area.<br>**3.** Number of fish larvae affects abundance of adult fish.<br>**4.** Turbidity affects predation success, reduces fitness & can cause fish to avoid extraction area.
**Fish eggs**	**1.** Sedimentation may blanket eggs.<br>**2.** Harmful substances may destroy eggs.
**Fish larvae**	**1.** Bottom sedimentation may smother larvae.<br>**2.** Underwater noise may reduce fitness.<br>**3.** Harmful substances may kill larvae.<br>**4.** Quantity of fish eggs affects larval abundance.

Functional groups of organisms	Affecting pressures or other variables
**Phytoplankton**	**1.** Sediment in the water column may shade phytoplankton.<br>**2.** Toxin release may modify community composition.<br>**3.** Nutrients increase primary production.
**Zooplankton**	**1.** Sediment in water column may clog feeding organs & impact individual fitness.<br>**2.** Quantity and quality of phytoplankton as food source affects zooplankton biomass.
**Pelagic crustacean**	**1.** Underwater noise induces stress and reduces fitness in organisms.<br>**2.** Toxic substances may reduce fitness.<br>**3.** Quantity and quality of mobile infauna as a food source affect biomass.<br>**4.** Habitat modification may affect recolonization capacity.
**Macrophytes**	**1.** Higher nutrient concentrations increase macrophytes growth.<br>**2.** Turbidity reduces light availability in the water column and changes the quality of light.<br>**3.** Toxic substances may affect macrophyte growth.<br>**4.** Sedimentation may smother macrophytes.
**Benthic microbes**	**1.** Removal of specific taxa through concretion removal.<br>**2.** Nutrient release from sediment affects growth.
**Organisms attached to nodules**	**1.** Concretion extraction removes organisms.<br>**2.** Sediment plume may clog feeding organs & blanket organisms
**Sessile epifauna filter/suspension feeder**	**1.** Noise may induce stress & reduce fitness.<br>**2.** Concretion extraction removes organisms.<br>**3.** Sediment plume may clog and blanket organisms.<br>**4.** Low oxygen concentrations reduce fitness.<br>**5.** Toxic substances reduce fitness.

Functional groups of organisms	Affecting pressures or other variables
**Mobile infauna filter/suspension / deposit feeder**	**1.** Noise may induce stress.<br>**2.** Concretion extraction removes organisms.<br>**3.** Habitat change affects recovery<br>**4.** Sediment plume may clog feeding organs.<br>**5.** Low oxygen concentrations reduce fitness.<br>**6.** Toxic substances reduce fitness.
**Mobile epifauna filter / suspension feeder**	**1.** Noise may induce stress<br>**2.** Concretion extraction removes organisms.<br>**3.** Habitat change affects recovery<br>**4.** Sediment plume may blanket<br>**5.** Low oxygen concentrations reduce fitness.
**Mobile epifauna deposit-feeder (slow-moving)**	**1.** Noise may induce stress.<br>**2.** Concretion extraction removes organisms.<br>**3.** Habitat change affects recovery.<br>**4.** Sediment plume may blanket organisms.<br>**5.** Low oxygen concentrations reduce fitness.<br>**6.** Toxic substances reduce fitness.
**Mobile epifauna predator (fast-moving)**	**1.** Noise may induce stress.<br>**2.** Habitat change affects recovery<br>**3.** Sediment plume may blanket organisms.<br>**4.** Low oxygen concentrations reduce fitness.<br>**5.** Toxic substances reduce fitness.

Table S5. Causal connections between the physicochemical pressures and other model variables.

Pressures from mineral extraction	Parameters affecting pressures
**Underwater noise**	**1.** Seafloor substrate type (hard/soft) affects noise levels.<br>**2.** Timing of activity (affects stratification in the water column).
**Concretion removal**	-
**Modification of seafloor substrate type**	**1.** Depth of extracted sediment.<br>**2.** Sediment dispersal at the bottom.
**Modification of seafloor topography**	**1.** Depth of extracted sediment
**Sediment dispersal in the water column**	**1.** Water column stratification<br>**2.** Surface currents
**Sediment dispersal at the bottom**	**1.** Depth of extracted sediment affects amount of sediment dispersed<br>**2.** Bottom currents affect sediment dispersal to neighboring areas.<br>**3.** type of extracted sediment
**Nutrient release from sediment**	1. nutrient concentrations in the sediment
**Nutrient release from pumping up bottom water**	**1.** Bottom water nutrient concentrations.<br>**2.** Volume of pumped water.

Table S6. Causal connection between other model variables.

Model variable	Factors affecting variable
**Turbidity in the euphotic zone**	**1.** Sediment dispersal in the water column increases turbidity
**Turbidity at the bottom**	**1.** Sediment dispersal in the water column<br>**2.** Sediment dispersal at the bottom.
**Sediment concentration at the bottom**	**1.** Sediment dispersal at the bottom.
**Change in habitat type**	**1.** Concretion removal<br>**2.** Modification of seafloor substrate type<br>**3.** Sediment dispersal at the bottom.
**Borrow holes**	**1.** Depth of extracted sediment affects creation of borrow holes.
**Seafloor erosion**	**1.** Concretion removal increases seafloor erosion when the hard substrates and cover is removed.
**Nutrient increase in water**	**1.** Nutrient release from sediment<br>**2.** Nutrient release from pumping up bottom water.

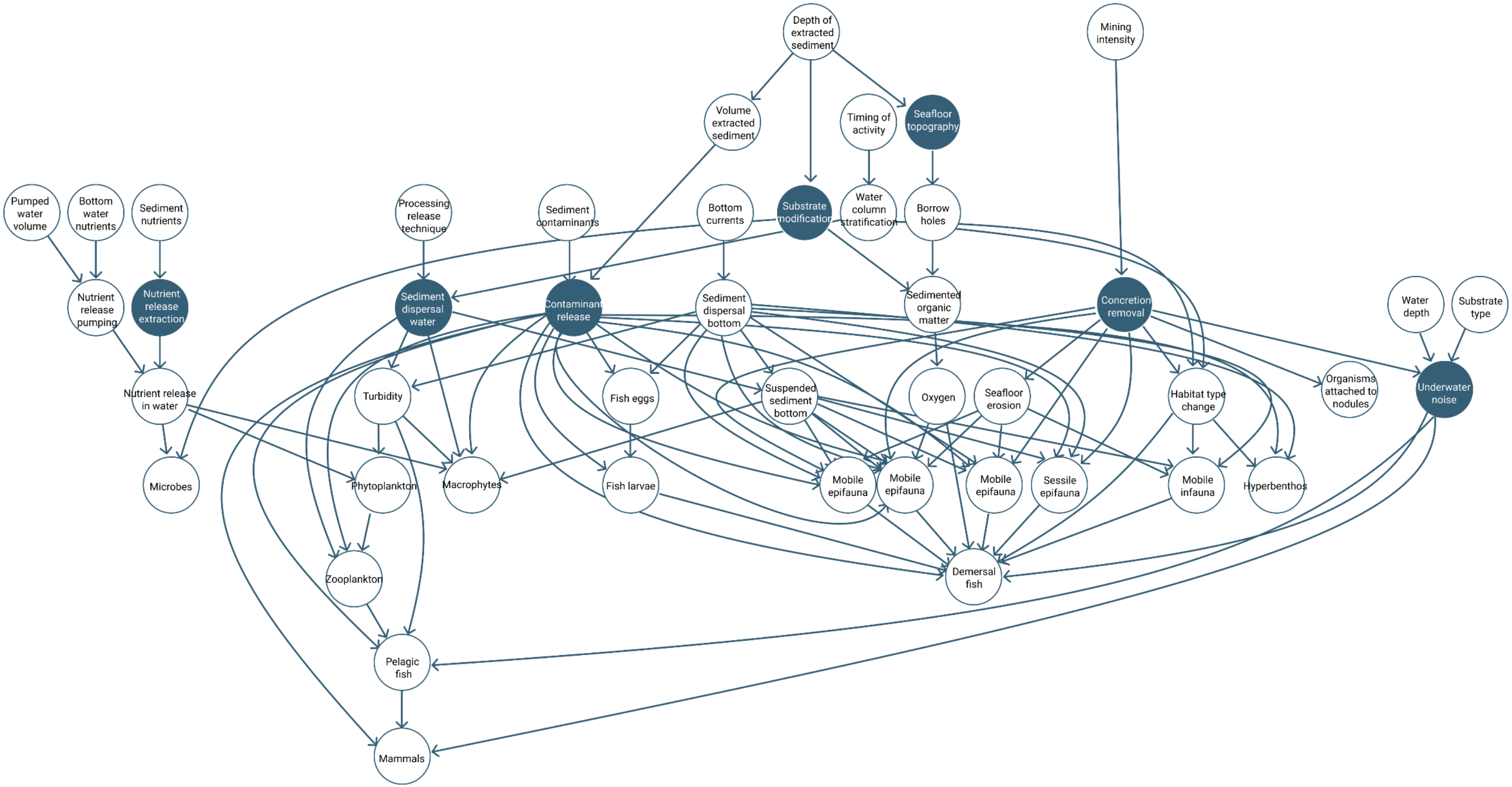

Figure S7. Combined causal map of the environmental and ecological effects of seabed nodule extraction on Baltic Sea ecosystem. The colored ovals denote pressures that were the starting point for each interview and the subsequent causal mapping.

Table S8. Spatiotemporal extent of the stressors.

**Stressor**	**Spatial extent**	**Temporal extent**
Habitat loss	Local	Long-term
Sediment deposition	Local to Regional	Transient to Long-term
Sediment substrate type	Local	Long-term
Suspended sediment	Local to Regional	Transient
Contaminant release	Local to Regional	Long-term
Nutrient increase	Regional	Transient
Underwater noise	Regional	Transient
